# Stem-like CD8+ T cells preserve HBV-specific responses in HBV/HIV co-infection

**DOI:** 10.1136/gutjnl-2025-335461

**Published:** 2025-12-04

**Authors:** Anucha Preechanukul, Aljawharah Alrubayyi, Bo Sun, Edward Arbe-Barnes, Jonida Kokici, Frances Gorou, Sarun Prasitdumrong, Kelly A S da Costa, Natasha Fisher-Pearson, Noshin Hussain, Stephanie Kucykowicz, Indrajit Ghosh, Fiona Burns, Sabine Kinloch, Pedro Simoes, Sanjay Bhagani, Patrick T F Kennedy, Mala K Maini, Rachael Bashford-Rogers, Upkar S Gill, Dimitra Peppa

**Affiliations:** 1 Institute of Immunity and Transplantation, University College London, London, UK; 2 Nuffield Department of Clinical Medicine, University of Oxford, Oxford, UK; 3 Nuffield Department of Clinical Neurosciences, University of Oxford, Oxford, UK; 4 Mortimer Market Clinic, Central Northwest London NHS Foundation Trust, London, UK; 5 Department of HIV Medicine, Royal Free London NHS Foundation Trust, London, UK; 6 Institute for Global Health, University College London, London, UK; 7 Barts Liver Centre, Barts & The London School of Medicine & Dentistry, Queen Mary University of London, London, UK; 8 Department of Biochemistry, University of Oxford, Oxford, UK

**Keywords:** CELLULAR IMMUNITY, CHRONIC VIRAL HEPATITIS, HEPATITIS B, IMMUNE RESPONSE, IMMUNOLOGY

## Abstract

**Background:**

Chronic hepatitis B virus (HBV) infection disproportionately affects people living with HIV, who are often excluded from functional cure studies.

**Objective:**

This study investigates CD8^+^ T cell profiles in HBV mono-infection versus HBV/HIV co-infection, examining the impact of long-term therapy on virus-specific responses to inform therapeutic strategies for immune restoration.

**Design:**

We analysed CD8^+^ T cell responses in 61 participants (HBV n=20, HBV/HIV n=20, HIV n=21), on suppressive antiviral therapy, assessing transcriptomic and proteomic profiles, focusing on exhaustion markers alongside virus-specific functional capabilities.

**Results:**

Transcriptomic analysis revealed distinct signatures in co-infection, with upregulation of TCR signalling genes, inhibitory pathways and progenitor-exhausted markers (*XCL2, TCF7, PDCD1, IL7R*). This profile scored highly for a precursor exhausted (Tpex) CD8^+^ T cell signature, reflecting stemness that maintains plasticity despite chronic antigen exposure. Proteomic analysis confirmed higher frequencies of Tpex (TCF-1^+^CD127^+^PD-1^+^) CD8^+^ T cells in co-infection, while HBV mono-infection showed predominance of terminally exhausted Tox^high^TCF-1^-^CD127^-^ cells. Tpex enrichment extended to HBV-specific populations corresponding with more robust, polyfunctional HBV-specific responses in co-infection against surface and core antigens. HBV-specific CD8 T cells maintained enhanced proliferative capacity and checkpoint responsiveness to anti-PDL1 blockade compared with HBV mono-infection. While co-infection was characterised by lower HBsAg levels and longer treatment duration, these factors alone did not account for the distinct immunological profiles.

**Conclusions:**

People with well-controlled HBV/HIV co-infection maintain robust CD8^+^ T cell responses with preserved stem-like properties supporting antiviral function. These results challenge assumptions about additive immune dysfunction in dual chronic infections and highlight the need for tailored immune-modulatory therapies.

**WHAT IS ALREADY KNOWN ON THIS TOPIC**

- Chronic hepatitis B virus (HBV) infection is marked by a progressive dysfunction of CD8^+^ T cells, which are crucial for antiviral responses. Traditionally, these responses were thought to be more severely impacted in people with HBV/HIV co-infection.

**WHAT THIS STUDY ADDS**

- Our study provides new insights into the heterogeneous functional profiles of HBV-specific CD8^+^ T cells in people with HBV and HBV/HIV co-infection in the current antiretroviral therapy era.
- People living with HBV/HIV co-infection suppressed on antivirals have a higher prevalence of precursor exhausted CD8^+^ T cells (Tpex), alongside more effective antiviral responses when compared with those with HBV mono-infection.
- Our data demonstrate intrinsic differences in T cell profiles, revealing a paradoxical increase in terminally exhausted CD8^+^ T cells in people with HBV mono-infection.

**HOW THIS STUDY MIGHT AFFECT RESEARCH, PRACTICE OR POLICY**

- By providing a clearer understanding of CD8^+^ T cell dynamics in HBV mono-infection and HBV/HIV co-infection, our findings could inform the design of tailored immunotherapies aimed at revitalising antiviral responses.
- Furthermore, this research may influence practices regarding clinical management emphasising the need for early intervention strategies and recognition that T cell exhaustion must be interpreted within the context of infection status.
- Identify people with HBV/HIV co-infection as potential more suitable candidates for check-point based immunotherapies.

## Introduction

 Chronic hepatitis B virus (cHBV) infection remains a significant health challenge, particularly among people living with HIV, where approximately 10% are affected by concurrent cHBV.^1^ Yet people with co-infection are traditionally excluded from clinical trials, and their immune responses/HBV-specific immunity remain critically under-investigated, especially in the era of potent HBV-active antiretroviral therapies (tenofovir disoproxil fumarate (TDF)/tenofovir alafenamide). These responses are important to elucidate in order to develop new and safe immunotherapeutic strategies and guide their involvement in the HBV-cure agenda.

The pathogenesis of cHBV infection is influenced by immune responses, particularly those mediated by CD8^+^ T cells, which are instrumental for viral control and are thus promising targets for immunotherapeutic approaches aimed at achieving a functional cure (FC) (HBsAg loss) for cHBV infection.^2^ cHBV results in diminished CD8^+^ T cell responses characterised by variable degrees of functional impairment. Studies examining HBV-specific T cells demonstrated upregulation of inhibitory molecules, altered transcriptional profiles and metabolic dysfunction, reflecting a state of exhaustion due to prolonged antigen persistence.^3^,^5^ The complexity of HBV-induced dysfunction is highlighted by recent work showing that global CD8^+^ T cell exhaustion profiles can predict the restoration capacity of HBV-specific responses,^6^ distinct metabolic requirements exist between exhausted and functional virus-specific cells,^7^ and prolonged HBsAg exposure progressively impairs virus-specific T cells.^8^ These diverse T cell alterations highlight the multifaceted nature of immune dysfunction in cHBV. HIV co-infection is thought to exacerbate these functional deficits, leading to more pronounced dysregulation of HBV-specific CD8^+^ T cell responses due to the combined influence of HIV and HBV.^9^ In early studies, people with untreated co-infection had substantially fewer and narrower responses to HBV peptide stimulation compared with those with HBV mono-infection.^10^ Although limited research examining responses to antiviral therapy prior to the introduction of highly active antiretroviral therapy (HAART) indicated a modest recovery of HBV immune responses, these responses remained suboptimal.^11^ Notably, people with co-infection are more likely to achieve a FC following the introduction of ART^12 13^; however, the effects of long-term viral suppression on reconstitution of HBV-specific responses remain unknown. Our recent findings have indicated that, in the context of long-term therapy, people with HBV/HIV co-infection exhibit more favourable immunological profiles, evidenced by better preserved natural killer (NK) cell responses and lower levels of HBsAg and peripheral surrogates of HBV activity compared with those with HBV mono-infection.^14^ Given the critical role of CD8^+^ T cells in HBV control, there is an urgent need for increased insights into the specific profiles of CD8^+^ T cells in people with HBV/HIV.

Recent studies have revealed that exhausted CD8^+^ T cell populations are not homogeneous; they exhibit developmental and functional diversity, including the presence of stem-like progenitors expressing T cell factor 1 (TCF-1).^15^ These progenitors or precursors of exhausted CD8^+^ T cells (Tpex) retain the potential to mount effective immune responses,^16^ allowing for the possibility of therapeutic intervention aimed at restoring immune efficacy.^17^

Importantly, the nature of T cell dysfunction in cHBV may differ from other chronic infections. Emerging evidence suggests that T cell dysfunction in cHBV infection incorporates traits more reminiscent of a state of tolerance or anergy,^18^ underscoring the need for a more nuanced understanding of these dynamics, particularly in the under-explored context of co-infection.

In this study, we aimed to address these critical gaps in understanding the immune responses of individuals with HBV/HIV co-infection compared with those with HBV mono-infection in the current ART era. We hypothesised that earlier initiation and long-term viral suppression with potent ART might preserve functional CD8^+^ T cell responses in co-infection, contrary to traditional expectations of more severe immune dysfunction. We performed a detailed analysis at the single-cell level, complemented by assessments of functional virus-specific responses, to enhance our knowledge of the immunological landscape in these patient populations.

## Materials and methods

### Patient populations

A total of n=20 patients with HBV/HIV, n=20 people with HBV and n=21 with HIV were recruited at Mortimer Market Centre for Sexual Health and HIV research, the Ian Charleson Day Centre at the Royal Free Hospital (London, UK) or The Royal London Hospital (London, UK). All participants were negative for HCV infection and confirmed human cytomegalovirus (HCMV) seropositive. PBMCs, plasma and serum were collected as part of this study. Additional demographic and clinical information can be found in online supplemental table 1.

### Single cell sequencing

#### Library preparation and sequencing

PBMCs with >96% viability were used.^14^ Library construction was performed using the 10x next GEM Chips Chromium single cell library 5’ construction kit (10x Genomics, Pleasanton) as per the manufacturer’s protocol. Briefly, a maximum of 3000 cells were combined with barcoded single cell VDJ gel beads, cells and partitioning oil onto Chromium Next GEM Chip K. The resulting 10x Barcoded, full-length cDNA was recovered and amplified overnight via PCR with primers against common 5’ and 3’ ends added during the GEM reverse transcription process. Resulting cDNA was purified using SPRIselect beads (Beckman Coulter, High Wycombe, UK). The amount and quality of cDNA was determined using TapeStation (Agilent, UK). Purified libraries were analysed by Novogene. Donor characteristics analysed by single-cell RNA-sequencing (scRNA-seq) data are described in online supplemental table 2.

#### scRNA-seq preprocessing and procedures

scRNA-seq preprocessing and downstream analyses for this study have been previously described in detail.^14^ Briefly, we excluded any cell barcodes with fewer than 200 detected transcripts or more than 10% mitochondrial-encoded genes, retaining only cells expressing fewer than 3000 genes. We considered genes if they were expressed in at least five cells. After confirming that our data and the Azimuth PBMC reference atlas^19^ showed similar distributions of transcripts and gene counts, we demultiplexed hash-tagged samples using the Scanpy external implementation of Hashsolo^20^ and excluded doublets using Solo. For dataset integration, raw counts from the top 3000 highly variable genes were used to train a scVI model, which generated 20 latent dimensions for embedding. We then computed a nearest neighbour graph on these dimensions for cell clustering via iterative Leiden clustering at increasing resolutions to capture both coarse and fine cell type heterogeneity. Automated cell type annotations were performed with SingleR,^21^ followed by manual validation based on marker gene expression and hierarchical clustering.

Differential gene expression was calculated with Seurat’s FindMarkers function (using DESeq2 as the statistical backend), controlling for variable sample sizes across study groups. Functional enrichment analysis was carried out using the XGR package for pathway-level assessment and GSEApy for gene set enrichment analysis.^22^ Differential abundance analysis was performed with the R implementation of Milo.^23^ For the Milo buildGraph function, k was set to 40, and all 128 dimensions of the DRVI latent embedding were used. Where described, gene set scores were calculated with Scanpy’s score_genes function. The Tpex-score genes were derived from Zheng *et al*.^24^ For the unbiased gene programme discovery analysis, DRVI was implemented with default parameters.^25^ Each identified DR programme and its associated biological functions are detailed in online supplemental table 3.

#### Ex vivo phenotypic analysis of CD8^+^ T cells

The fluorochrome-conjugated antibodies utilised are detailed in online supplemental table 4. Briefly, cryopreserved PBMCs were thawed and allowed to rest at 37°C in a complete RPMI medium (Penicillin-Streptomycin, L-Glutamine, HEPES, non-essential amino acids, 2-Mercaptoethanol and 10% fetal bovine serum (FBS)). The cells were washed, resuspended in PBS and incubated for 20 min at 4°C with various antibody combinations along with a fixable live/dead stain (Invitrogen). The cells were subsequently fixed and permeabilised to facilitate the detection of intracellular antigens. For the identification of intranuclear markers, the Foxp3 intranuclear staining buffer kit (eBioscience) was used according to the manufacturer’s protocol. Total PBMCs were stained with APC-labelled HBV (core 18-27, envelope 183-191, envelope 335-343, and envelope 348-357), HCMV (pp65 495-504) and HIV (Gag 77-86) dextramers (Immudex) at 37°C for 15 min in complete RPMI with 10% FBS. After pelleting, cells were further stained as above. Dextramer staining was deemed positive if a distinct population (>0.02%) was identified. Data acquisition was conducted on a BD Fortessa X20 via BD FACSDiva 8.0 (BD Bioscience), and the resulting data analysis was carried out using FlowJo V.10 (TreeStar). The gating strategy for identifying CD8^+^ T cells is illustrated in online supplemental figure 2A. Stochastic neighbour embedding (SNE) and FlowSOM analysis was performed on the MRC Cytobank platform to visualise high-dimensional data. Flow cytometry files from all individuals within each study group were concatenated, and analysis was performed on 10 parameters (CCR7, CD45RA, CD127, TCF-1, HLA-DR, CD38, Granzyme B, Tim-3, PD-1 and TOX) using 20 000 equally sampled events per sample. FlowSOM was set to generate 100 clusters grouped into 10 metaclusters.

#### Functional assessment of virus-specific CD8^+^ T cells

Intracellular cytokine staining (ICS) was performed as previously described.^26^ PBMCs were thawed and allowed to rest at 37°C in complete RPMI medium with 5% CO_2_. PBMCs were then stimulated for 12–16 hours using 3 µg/mL of overlapping peptide pools from HBV (large envelope and capsid), HIV-1 Gag or positive control peptide pools derived from cytomegalovirus (HCMV), Epstein-Barr virus and influenza virus (CEF pool) (online supplemental table 5), alongside 0.005% DMSO as a negative control. This was done in the presence of αCD28/αCD49d co-stimulatory antibodies (1 µg/mL), Brefeldin A (eBioscience). Where indicated, 1 µg/mL anti-PD-L1 (Invitrogen) was added at the time of peptide stimulation. Poststimulation, cells were stained with anti-CCR7 at 37°C for 30 min, then surface stained at 4°C for 20 min along with a live/dead stain. Following fixation and permeabilisation, intracellular cytokines (IFN-γ APC, CD154 PE-Cy7, TNF-α FITC, IL-2 PerCP-eFluor 710) were assessed. Data acquisition was performed using a BD Fortessa X20, and analysis was conducted using FlowJo 10. Virus-specific CD8^+^ T cells were identified as those expressing combinations of IFN-γ, TNF-α and IL-2. A detailed list of antibodies used is found in online supplemental table 4.

#### Proliferation assay

Cryopreserved PMBCs were thawed, washed with 1×PBS and labelled with CellTrace Violet (CTV, Life Technologies) at a final concentration of 2.5 µM for 20 min at 37°C. The reaction was quenched with cold FBS, and cells were washed and resuspended in RPMI supplemented with 10% human AB serum (Sigma), 1 mM Penicillin/Strepomycin and 2 mM L-Glutamine. CTV-labelled PBMC were seeded at 2.5×10^5^ in 96-well round-bottom plates and stimulated with 1 µg/mL overlapping peptide pools spanning HBV large envelope or capsid proteins. Control conditions included media with 0.005% DMSO (Sigma) (negative control matching peptide pool vehicle) and 2 µg/mL CEF peptide pool (positive control). Cells were cultured for 7 days at 37 °C with 5% CO_2_ in a humidified incubator. Culture medium was refreshed at day 4 by replacing half the volume with fresh complete medium. Cells were harvested on day 7, stained with viability dye and surface/intranuclear markers as described above and analysed by flow cytometry. Proliferation was assessed by CTV dilution in viable CD8^+^ T cells compared with unstimulated controls.

### Statistical methods

Prism V.9 (Graphpad, San Diego) and R (URL https://www.R-project.org/) were used for statistical analysis. The Mann-Whitney U-test was used for single comparisons of groups, and the Wilcoxon paired t-test was used to compare two paired groups. Correlation analysis was carried out with non-parametric Spearman correlation. Kruskal-Wallis testing followed by Dunn test was used for pairwise comparison between gene module scores. The statistical significance is indicated in the figures. (*p<0.05, **p<0.01, ***p<0.001 and ****p<0.0001).

## Results

### Single-cell analysis of CD8^+^ T cells shows reduced cytotoxicity gene expression and enriched TCR signalling pathways in HBV/HIV co-infection

**Figure 1:**
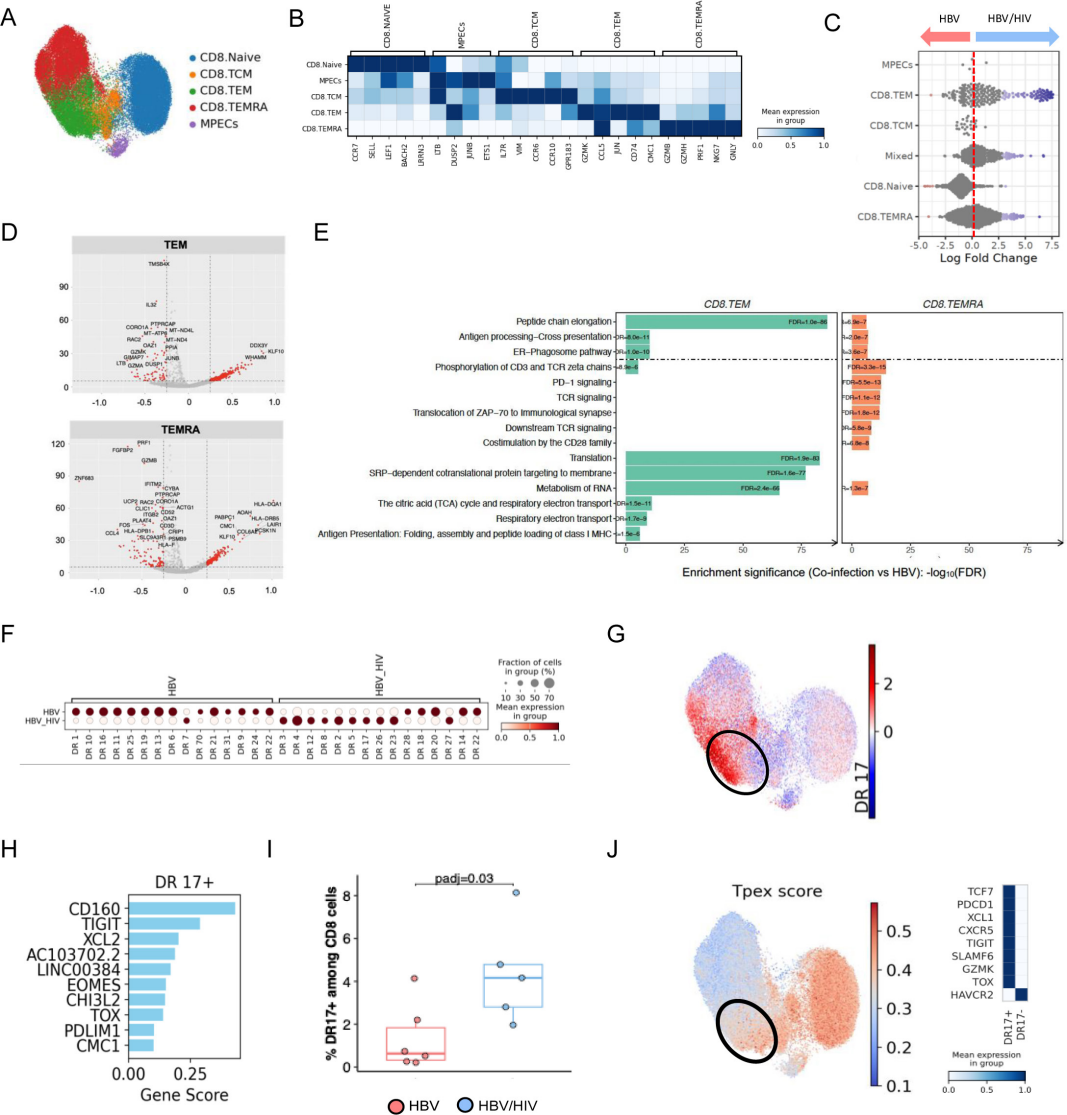
Transcriptomic profiles of CD8^+^T cell subsets in HBV mono-infection versus HBV/HIV co-infection. (**A**) UMAP embedding of n=51 843 CD8^+^ T cells from n=11 individuals (HBV n=6; HBV/ HIV co-infection, n=5). (**B**) Expression heatmap showing distinct gene expression profiles for CD8^+^ T cell subsets. Genes were selected as the five most significant differentially expressed genes per cluster. (**C**) Milo cell neighbourhood differential abundance plots of the significantly enriched neighbourhoods between HBV mono-infection versus HBV/HIV co-infection (FDR<0.05). (**D**) Volcano plot of DESeq2 differential gene expression analysis; significant genes (FDR<0.05 and average log2FC >/< 0.25) are highlighted. The direction is in favour of HBV/HIV co-infection, for example, positive fold changes are upregulated in HBV/HIV co-infection versus HBV mono-infection. (**E**) Bar plot of most significantly enriched (FDR<0.005) reactome pathways in HBV/HIV co-infection versus HBV mono-infection. (**F**) Differential expression analysis of DRVI-derived gene programmes (denoted numerically as DRs). (**G**) UMAP embedding showing activity of DR17. (**H**) Top identified genes for DR17. (**I**) Percentage of CD8 T cells expressing DR17 in individuals with HBV mono-infection and HBV/HIV co-infection; statistical significance between groups was assessed by the two-sided Mann-Whitney U test with Benjamini-Hochberg correction (*padj=0.03*). (**J**) The activity of the Tpex score on UMAP. DR, dimensionality reduction; DRVI, disentangled representation variational inference; ER, endoplasmic reticulum; FDR, false discovery rate; HBV, Hepatitis B Virus; MHC, major histocompatibility complex; MPEC, memory precursor effector cells; UMAP, uniform manifold approximation and projection.

To explore the effects of HBV and combined effect of HBV/HIV on CD8^+^ T cell populations, we utilised available single-cell sequencing PBMC data (using 10X Chromium (5’ transcriptome) from n=5 HBV/HIV and n=6 HBV donors, predominately on TDF treatment (online supplemental table 2).^14^ To account for the effect of HCMV, all donors were confirmed human HCMV-seropositive. CD8^+^ T cell subsets—naive CD8^+^ T cell (TN), central memory (TCM) CD8^+^ T cell, effector memory (TEM) CD8^+^ T cell, terminally differentiated effector memory (TEMRA) CD8^+^ T cell and memory precursor effector cell, were identified by clustering based on their differentially expressed genes and visualised on a uniform manifold approximation and projection (figure 1A,B). Further validation of annotations was performed using correlation-based annotation with SingleR^21^ using the Monaco Immune dataset as reference^27^ (online supplemental figure 1A,B).

To gain insight into cellular differential abundance and compositional shifts between HBV mono-infection versus HBV/HIV co-infection, MiloR was used for differential abundance testing. While naive CD8^+^ T-cells trended towards more abundance in HBV mono-infection, we found that most of the significant changes driven by HBV/HIV co-infection were within the CD8^+^ TEM compartment (figure 1C). Comparing the transcriptional profiles of CD8^+^ TEM cells between patient groups, donors with co-infection showed an upregulation of *KLF10*, which encodes an effector protein of transforming growth factor beta (TGF-β) signalling,^28^ *DDX3Y*, which is involved in RNA binding and the formation of intramolecular interactions, and *WHAMM*, which encodes a protein that mediates membrane dynamics and cytoskeletal organisation within cells^29^ (figure 1D). In contrast, several cytotoxicity genes were upregulated in the CD8^+^ TEM and TEMRA clusters of mono-infected patients (figure 1D), correlating with higher cytotoxicity scores observed in these populations in HBV mono-infection (online supplemental figure 1C). Genes driving CD8^+^ T cell remodelling were reflected in differentially regulated gene pathways, with enrichment of TCR downstream signalling, co-stimulation, PD-1 signalling and TCA cycle and translation observed in the CD8^+^ T cell populations in the co-infection group (figure 1E).

Next, we sought to identify de novo gene programmes that might be differentially used across mono-infection and co-infection. To do so, we used DRVI^25^ (an unsupervised deep generative model that learns nonlinear, disentangled representations of single-cell omics data), allowing us to discover biologically meaningful latent dimensions and nonlinear gene programmes. Applying DRVI led to the identification of 39 gene programmes (online supplemental figure 1D,E; online supplemental table 3). Notably, ‘DR17’ was upregulated in the same TEM neighbourhood identified by MiloR analysis as being enriched in HBV/HIV co-infection (figure 1F,G).

Among the relevant genes enriched in DR17 were *CD160, TIGIT, XCL2*, *CHI3L2*, *EOMES*, *TOX*, *PDLIM1* and *CMC1* (figure 1H). While expression of individual DR17-associated genes varied modestly between groups (online supplemental figure 2A), the proportion of CD8 T cells classified as DR17+ was significantly elevated in co-infection (figure 1I), consistent with coordinated transcriptional programme usage rather than individual gene upregulation. Elevated expression of *CHI3L2* (involved in inflammation regulation)^30^ and *CMC1* (involved in mitochondrial function)^31^ suggests cellular and metabolic adaptations of CD8^+^ T cells in co-infection. Of particular interest, *XCL2* has been associated with progenitor or precursors of exhausted T cells (Tpex)^32^ and DR17 expressed a constellation of canonical Tpex markers (*TCF7*, *PDCD1*, *IL7R*, *GZMK*, *TOX*) while notably lacking *HAVCR2* (which encodes TIM-3, characterising terminally exhausted populations) (online supplemental figure 2B). In addition, coexpression of inhibitory receptors (*CD160*, *TIGIT*) alongside exhaustion-associated transcription factors (*EOMES*, *TOX*) supports a Tpex-like transcriptional programme.^17 33^ This phenotype aligns with a stem-like, progenitor-exhausted state associated with a favourable response to combination therapy in hepatocellular carcinoma.^32^ To quantitatively assess this, we calculated a Tpex signature score using gene sets derived from a pan-cancer single-cell atlas.^24^ As expected, DR17 scored highly for the Tpex signature alongside naïve and MPEC populations (figure 1J). This overlap likely reflects the shared transcriptional programmes, particularly genes related to ‘stemness’ and persistence that are common to both Tpex and naïve/memory-precursor CD8 T cells enabling functional plasticity under conditions of chronic antigen exposure. Overall, these data suggest that DR17 represents a functionally poised, stem-like CD8^+^ T cell state in HBV/HIV co-infection; capable of balancing inhibitory signalling with the capacity for self-renewal and effector differentiation.

### Precursors of exhausted CD8^+^ T cells are enriched in HBV/HIV co-infection

To explore whether the proteomic profile of CD8^+^ T cells in individuals with HBV mono-infection (n=20) and HBV/HIV co-infection (n=20) mirrors transcriptomic signatures, we conducted a phenotypic analysis within our wider cohort. Matched donors with HIV mono-infection (n=21) virally suppressed on ART were utilised as a comparator group (online supplemental table 1). Using a curated cytometry panel and gating strategy (online supplemental figure 3A), we evaluated CD8^+^ T cell subsets. As expected, higher frequencies of CD8^+^ T cells were observed in the context of HIV infection (online supplemental figure 3B). HBV/HIV co-infection and HIV mono-infection showed increased frequencies of CD8^+^ TEM and TEMRA, while HBV mono-infection predominantly exhibited CD8^+^ naive T cells, consistent with findings from single-cell analyses (online supplemental figure 3C,D).

**Figure 2:**
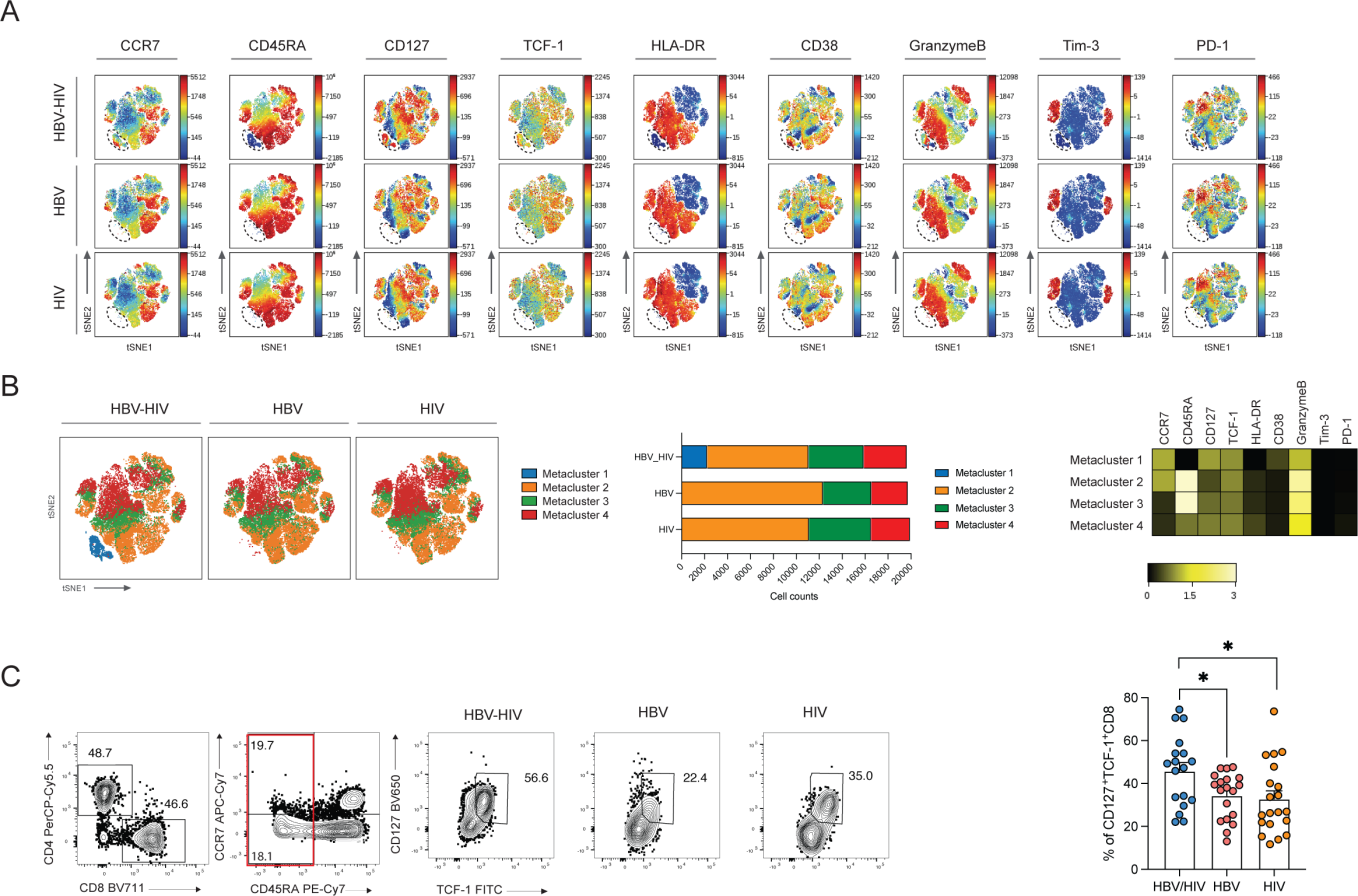
Stem-like CD8^+^ T cells are enriched in HBV/HIV co-infection. (**A**) viSNE analysis conducted on CD8^+^ T cells using concatenated files from HBV/HIV+ (n = 20), HBV+ (n = 20), and HIV+ (n=21) donors in each study group. Each point on the high-dimensional mapping represents an individual cell, and colour intensity represents the expression of selected markers. (**B**) viSNE map of FlowSOM metaclusters of CD8^+^ T cells from people with HBV/HIV, HBV or HIV. Metaclusters are colour-coded. Cell count of each FlowSOM metacluster out of total CD8^+^ T cells (20 000 cells/group). Heatmap of the selected markers for CD8^+^T cell clusters. (**C**) Frequency of CD127^+^TCF^-^1^+^CD45RA^+^CCR7^-/low^CD8^+^ T cells via traditional gating in all patient groups. Summary data of the proportion of CD127^+^TCF-1^+^CD45RA^+^CCR7^-/low^CD8^+^ T cells in the study groups. Significance determined by two-tailed Mann-Whitney U test. HBV, hepatitis B virus; t-SNE, t-distributed SNE; viSNE, visualisations stochastic neighbour embedding. *p<0.05.

Using an unbiased global t-distributed SNE (t-SNE) high-dimensional analysis and FlowSOM clustering, we further identified a distinct subpopulation (metacluster) that was more prominent in people with HBV/HIV co-infection relative to the other groups (figure 2A). This metacluster 1, which was more abundant in HBV/HIV co-infection, displayed a phenotypic signature characterised by the presence of CD45RA^-^CCR7^+/-^TIM3^-^PD1^+/-^CD127^high^TCF1^+^ (figure 2A–B). Manual gating analysis further confirmed increased frequencies of CD127^high^TCF1^+^ CM/TEM CD8^+^ T cells, which were particularly expanded within specific donors with HBV/HIV co-infection (figure 2B,C), suggesting a less differentiated memory-like state with preserved stemness properties.

**Figure 3:**
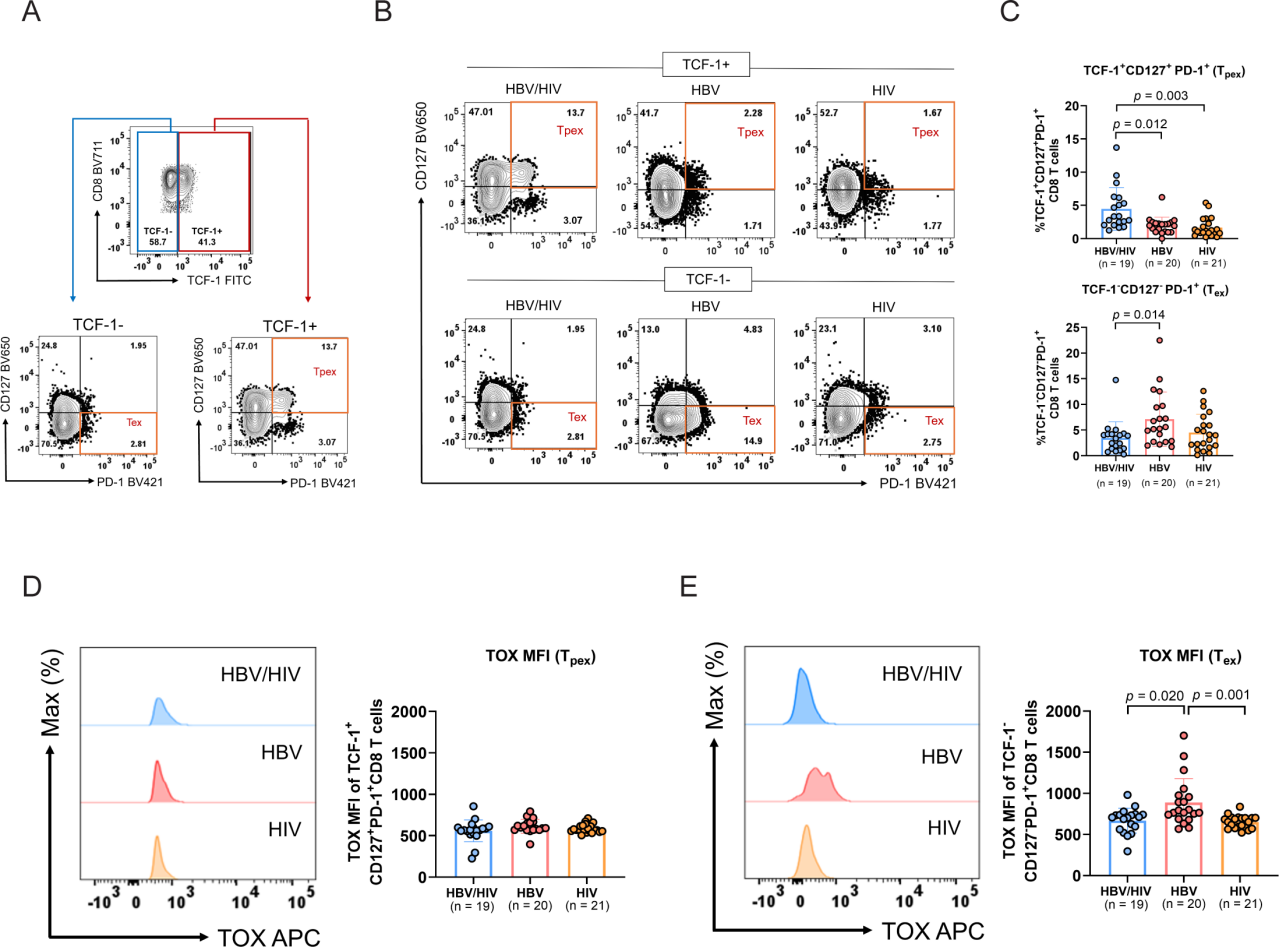
Phenotypic characterisation of Tpex and Tex CD8^+^ T cells in peripheral blood of donors with HBV/HIV, HBV and HIV infection. (**A**) Flow plots of gating strategy for identifying Tpex and Tex (**B**) Flow plots of Tpex and Tex profiles identified by expression of TCF-1, CD127 and PD-1 in peripheral blood and (**C**) summary data from HBV/HIV+ (n=19), HBV+ (n=20), and HIV+ (n=20) donors. Representative histogram and summary data of TOX MFI on (**D**) Tpex and (**E**) Tex CD8^+^ T cells from HBV/HIV+ (n=19), HBV+ (n=20), and HIV+ (n=20) donors. Bar charts show the median value with IQR and each dot represents one donor in a group. Statistical significance was assessed by Kruskal-Wallis with Dunn’s multiple comparison test. Representative flow plots show data from the donor with the highest response magnitude. DMSO, dimethyl sulfoxide; HBV, hepatitis B virus; MFI, mean fluorescence intensity.

Building on the transcriptomic findings and FlowSOM clustering, we characterised the exhaustion landscape in greater detail across the study groups, with particular attention to co-expression patterns of TCF-1, CD127 and PD-1, signatures associated with Tpex versus terminal exhaustion (Tex) states on global CD8^+^ T cells (figure 3A–C). While conventional exhaustion/activation markers (PD-1, HLADR/CD38) showed no significant differences between groups (online supplemental figure 4A,B), we observed reduced CD127 expression on CD8^+^ T cells in HBV mono-infection alongside elevated TOX levels (online supplemental figure 4C,D), robust expression of which results in commitment to Tex.^34^ Higher frequencies of CD8^+^ T cells with a stem-cell Tpex phenotype expressing TCF-1^+^CD127^+^PD-1^+^ were identified in people with HBV/HIV compared with either mono-infection group (figure 3B,C), mirroring the transcriptomic enrichment of stemness-associated genes in this population. Conversely, Tex CD8^+^ T cells characterised by TCF-1-CD127-PD-1+ expression were predominant in HBV mono-infection (figure 3B,C). While TOX expression was similar across Tpex populations in all groups (figure 3D), TCF-1^−^CD127^−^PD-1^+^TOX^+^ cells were significantly increased in HBV mono-infection (figure 3E), in keeping with a terminal exhaustion state. Tpex cells resided primarily within the TEM compartment, while Tex cells were enriched in TEMRA populations in HBV mono-infection (online supplemental figure 5A,B). To further address the differentiation stage of the particular CD127/TFC-1 subsets, we stained for the effector cell molecule granzyme-B. As expected, Tpex populations were further characterised by lower granzyme-B expression^35^; by contrast, granzyme-B^+^ Tex CD8^+^ T cells were predominant in HBV mono-infection (online supplemental figure 5C,D) aligning with the higher cytotoxicity signatures observed in the transcriptomic data.

Collectively, our findings reveal distinct CD8^+^ T cell exhaustion landscapes in HBV versus HBV/HIV co-infection.

### Higher frequency and polyfunctional HBV-specific CD8^+^ T cell responses in HBV/HIV co-infection compared with HBV

Having established distinct exhaustion profiles in global CD8^+^T cell populations, we next sought to determine whether these phenotypic differences translated to functional discrepancies in virus-specific CD8^+^T cell responses between individuals with HBV mono-infection and those with HBV/HIV co-infection. ICS was employed to investigate the composition and polyfunctionality of T cell responses among individuals with HBV/HIV co-infection, as well as those with HBV and HIV mono-infection. PBMCs were stimulated overnight using overlapping peptides targeting the HBV surface (S) and core proteins, alongside CEF and HIV-1 gag peptides within the same individuals.

**Figure 4:**
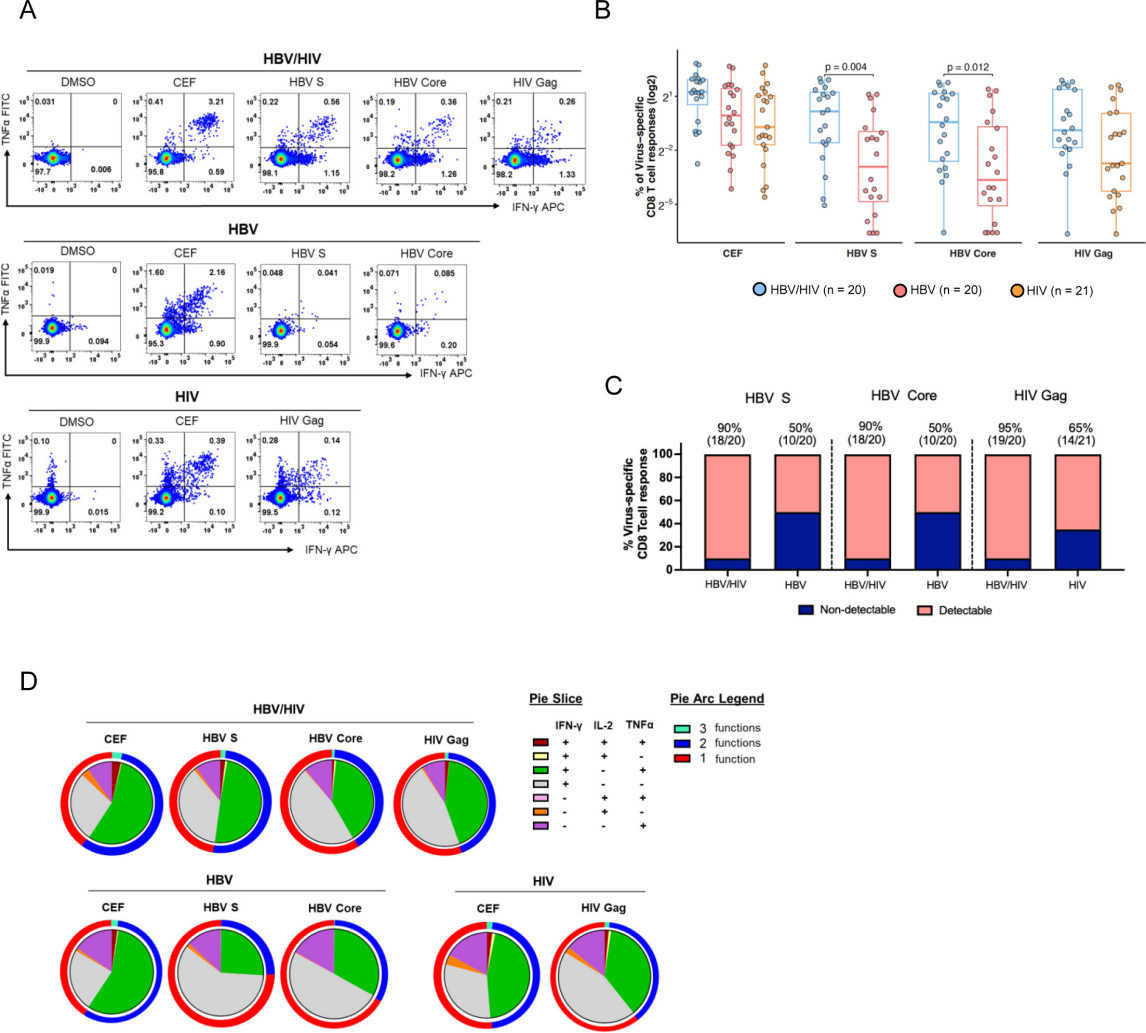
Composition of HBV and HIV-specific CD8^+^ T cells in HBV/HIV, HBV and HIV infection. Intracellular cytokine staining (ICS) was performed to examine virus-specific CD8^+^ T cells to the indicated peptide pools in HBV/HIV+ (n = 20), HBV+ (n = 20), and HIV+ (n=21) donors. (**A**) Representative flow cytometric plots for the identification of antigen-specific CD8^+^ T cells based on double expression (IFN-γ^+^TNF-α^+^) following overnight stimulation with DMSO in media (control), CEF pool (positive control) or overlapping HBV-S, HBV-core and HIV-gag peptides. (**B**) Summary of aggregated CD8^+^ T cell responses (IFN-γ^+^TNF-α^+^) against CEF, HBV-S, HBV-core or HIV-gag peptide pools from HBV/HIV+ (n = 20), HBV+ (n = 20), and HIV+ (n=21) donors. (**C**) Proportion of detectable and non-detectable virus-specific CD8^+^ T cell responses in HBV/HIV+, HBV+, and HIV+donors. (**D**) Pie charts representing the polyfunctional subsets of CEF-, HBV-S-, HBV-core- or HIV-gag-specific CD8^+^ T cell responses with IFN-γ, TNF-α and IL-2 expression, and pie arcs denoting the relative proportions of virus-specific CD8^+^T cell response for one (red), two (blue) and three (green) cytokines. Statistical significance was assessed by Kruskal-Wallis with Dunn’s multiple comparison test (p<0.05). HBV, hepatitis B virus.

Notably, after overnight stimulation, higher magnitude HBV-specific responses, identified by co-expression of IFN-γ and TNF-α, were seen in donors with HBV/HIV co-infection relative to donors with HBV (figure 4A,B). Over 90% of donors with co-infection demonstrated detectable HBV-specific responses (>0.05% IFN-γ^+^TNF-α^+^ CD8^+^ T cells), compared with 50% in HBV mono-infection (figure 4C). Within individual donors, HBV-specific responses were comparable to those against CEF or HIV-1 gag peptides (figure 4B). HIV-1 gag-specific responses were similar between people with HBV/HIV co-infection and those with HIV mono-infection (figure 4B). HBV-specific CD8^+^ T cells displayed polyfunctional responses in people with co-infection, particularly against HBV surface protein (figure 4D). HBV-specific CD8^+^ T cells primarily displayed EM/TEMRA phenotypes, with core-specific responses in people with co-infection also exhibiting a more notable CM phenotype compared with HBV mono-infection (online supplemental figure 6A,B). While people with co-infection showed higher mean frequencies of CEF responses compared with those with mono-infection, these differences were not statistically significant and displayed similar polyfunctional profiles (figure 4A,B,D). Despite elevated PD-1 expression on both HBV-specific and HIV-specific CD8^+^ T cells compared with CEF-specific cells (online supplemental figure 6C,D), this did not compromise their functional capacity, suggesting PD-1 expression in this context may not exclusively indicate exhaustion.

**Figure 5:**
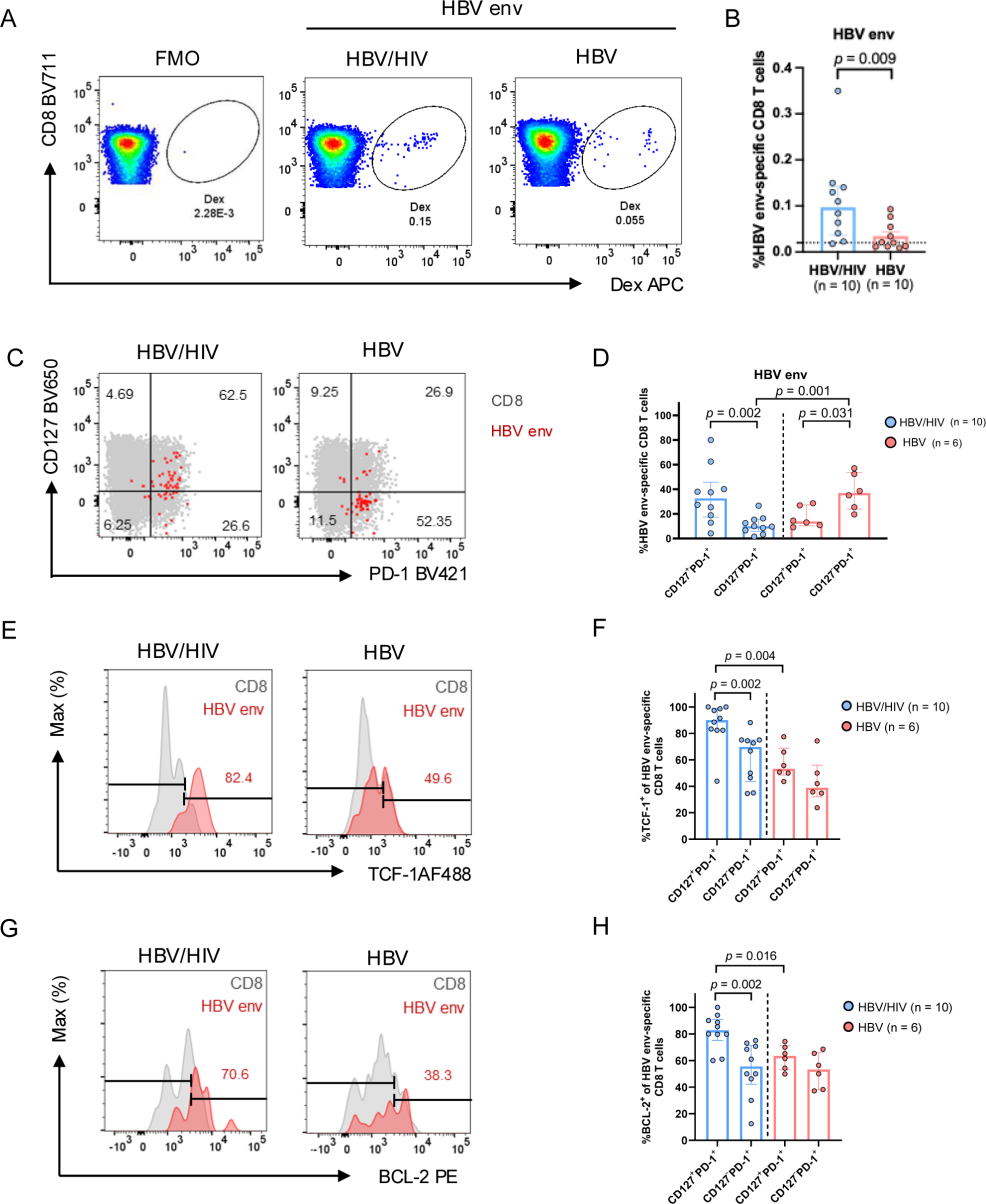
Ex vivo HBV-specific CD8^+^T cells in HBV/HIV co-infection. (**A**) Representative flow plots and (**B**) summary data of HBV_env_–specific CD8^+^ T cell identification by dextramer staining in HBV/HIV and HBV donors n=10 per group. (**C**) Representative flow plots (red: HBV_env_–specific CD8^+^ T cell; grey: corresponding global CD8^+^ T cells) and (**D**) summary data of CD127/PD-1 co-expression analysis of HBV_env_-specific CD8^+^ T cells derived from individuals with HBV/HIV and HBV with detectable responses. (**E**) Representative histogram and gating of TCF-1 expression on HBV_env_-specific CD8^+^ T and global CD8^+^ T cells and summary data (**F**) of TCF-1 expression on HBV_env_-specific CD8^+^ T with respect to CD127/PD-1 subsets. (**G**) Representative histogram and gating of BCL-2 expression on HBV_env_-specific CD8^+^ T and global CD8^+^ T cells and (**H**) summary data of BCL-2 expression on HBV_env_-specific CD8^+^ T with respect to CD127/PD-1 subsets. Representative histograms of the individual markers are depicted (grey: corresponding global CD8^+^ T cells and red: HBV epitope-specific CD8^+^ T cells). Bar charts show the median value with IQR. Statistics were performed using a two-tailed Wilcoxon matched-pairs signed-rank test (p<0.05). Representative examples show data from donors with the highest response magnitude. FMO, fluorescence minus one; HBV, hepatitis B virus.

To further characterise HBV-specific CD8^+^ T cells, we employed major histocompatibility complex (MHC) I dextramers to detect virus-specific CD8^+^ T cells directly ex vivo targeting known HLA-A*02-restricted epitopes. We focused on HBV_env_ (combined env_183–191_, env_335–343_, and env_348–357_), pp65_495-504_ and Gag_77-86_ in a subset of HLA-A*02-positive donors from both co-infection and HBV mono-infection groups. HBV-env-specific CD8^+^ T cells were more frequently detected in people with co-infection (figure 5A,B). Phenotypic analysis showed that HBV env-specific CD8^+^ T cells from donors with co-infection displayed higher frequencies of Tpex cells (PD-1^+^/CD127^+^) compared with CD127-PD1+cells whereas the inverse pattern was observed in people with HBV mono-infection (figure 5C,D). In patients with co-infection, HCMV pp65-specific and Gag-specific CD8^+^ T cells similarly showed an enrichment of CD127^+^PD-1^+^ (Tpex phenotype) compared with CD127^-^PD-1^+^ (Tex phenotype) (online supplemental figure 7A,B). Next, we assessed the expression of the transcription factor TCF-1 and anti-apoptotic molecule BCL-2 that have been shown to best determine the memory-like phenotype and cellular persistence of this subset.^36^ CD127^+^PD-1^+^ HBV env-specific CD8^+^ T cells contained higher mean frequencies of TCF-1^+^ and BCL-2^+^ cells compared with CD127^-^PD-1^+^ populations in people with co-infection compared with HBV mono-infection (figure 5E–H). Within donors with co-infection, HCMV-specific and HIV Gag-specific cells showed similar patterns with higher expression of TCF-1 and BCL-2 in CD127+PD1+ compared with to CD127-PD1+populations (online supplemental figure 7C,D). These results would be in keeping with the enhanced survival potential of this precursor exhausted subset.

### Enhanced proliferation and anti-PD-L1 responsiveness of HBV-specific CD8^+^ T cells in HBV/HIV co-infection

**Figure 6:**
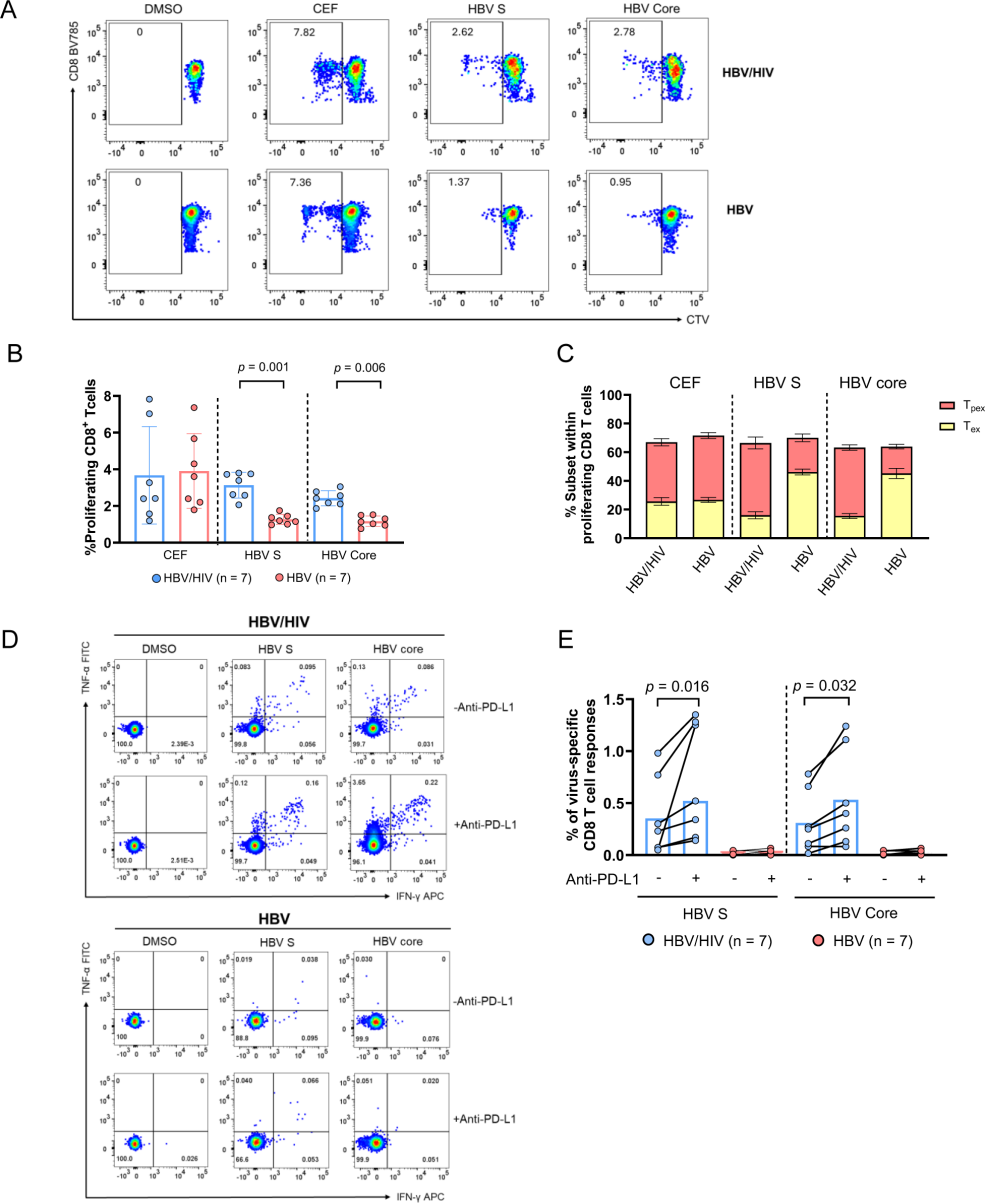
Proliferative responses in CD8^+^ T cells to HBV antigens and restoration of HBV-specific response by anti-PD-L1 blockade in people living with HBV/HIV and HBV. (**A**) Flow cytometric plots and (**B**) summary data of proliferating cells in response to peptide pool stimulation in HBV/HIV+ (n = 7), HBV+ (n = 7) volunteers in CD8^+^ T cells DMSO (negative control), CEF pool (positive control) or overlapping peptide pools spanning HBV S and core. (**C**) summary data of Tpex and Tex subsets within proliferating CD8^+^ T cells in response to CEF, HBV S and HBV core in individuals with HBV/HIV and HBV. (**D**) Flow cytometric plots and (**E**) summary data of aggregated CD8 T-cell responses (IFN-γ^+^TNF-α^+^) against HBV S and HBV core in the presence or absence of PD-L1 blockade in HBV/HIV+ (n=10) and HBV+ (n=10) donors. Statistics were performed using a two-tailed Wilcoxon matched-pairs signed-rank test (p<0.05). Representative examples show data from donors with the highest response magnitude. DMSO, dimethyl sulfoxide; HBV, hepatitis B virus.

To determine whether the preserved Tpex phenotype corresponded with maintained proliferative function, we assessed HBV-specific CD8^+^ T cell responses using CTV dilution assays. People with HBV/HIV co-infection demonstrated significantly higher frequencies of proliferating HBV-specific CD8^+^ T cells following peptide stimulation compared with those with HBV mono-infection (figure 6A,B). This enhanced proliferative capacity—a defining characteristic of Tpex cells^17^—was observed for both HBV surface (p=0.001) and core (p=0.006) antigens. Analysis of the proliferating cells showed that HBV-specific responses in people with co-infection maintained a predominant Tpex phenotype, contrasting with the Tex profile in HBV mono-infection (figure 6C). CEF-specific responses showed comparable Tpex/Tex distributions between groups, in keeping with selective Tpex enrichment to HBV responses in people with co-infection (figure 6C).

Notably, anti-PD-L1 blockade during overnight peptide stimulation enhanced HBV-specific CD8^+^T cell responses in HBV/HIV patients (figure 6D,E), with significant increases observed for both surface (p=0.016) and core (p=0.032) antigens. This checkpoint responsiveness was specific to people with co-infection, as those with HBV mono-infection showed minimal responses to anti-PD-L1 (figure 6D,E). These functional assays demonstrate that the stem-like CD8^+^ T cells identified in co-infection retain both their proliferative potential and capacity to respond to checkpoint blockade, highlighting their therapeutic relevance.

### Lower HBsAg levels and longer treatment duration distinguish HBV/HIV co-infection

**Figure 7:**
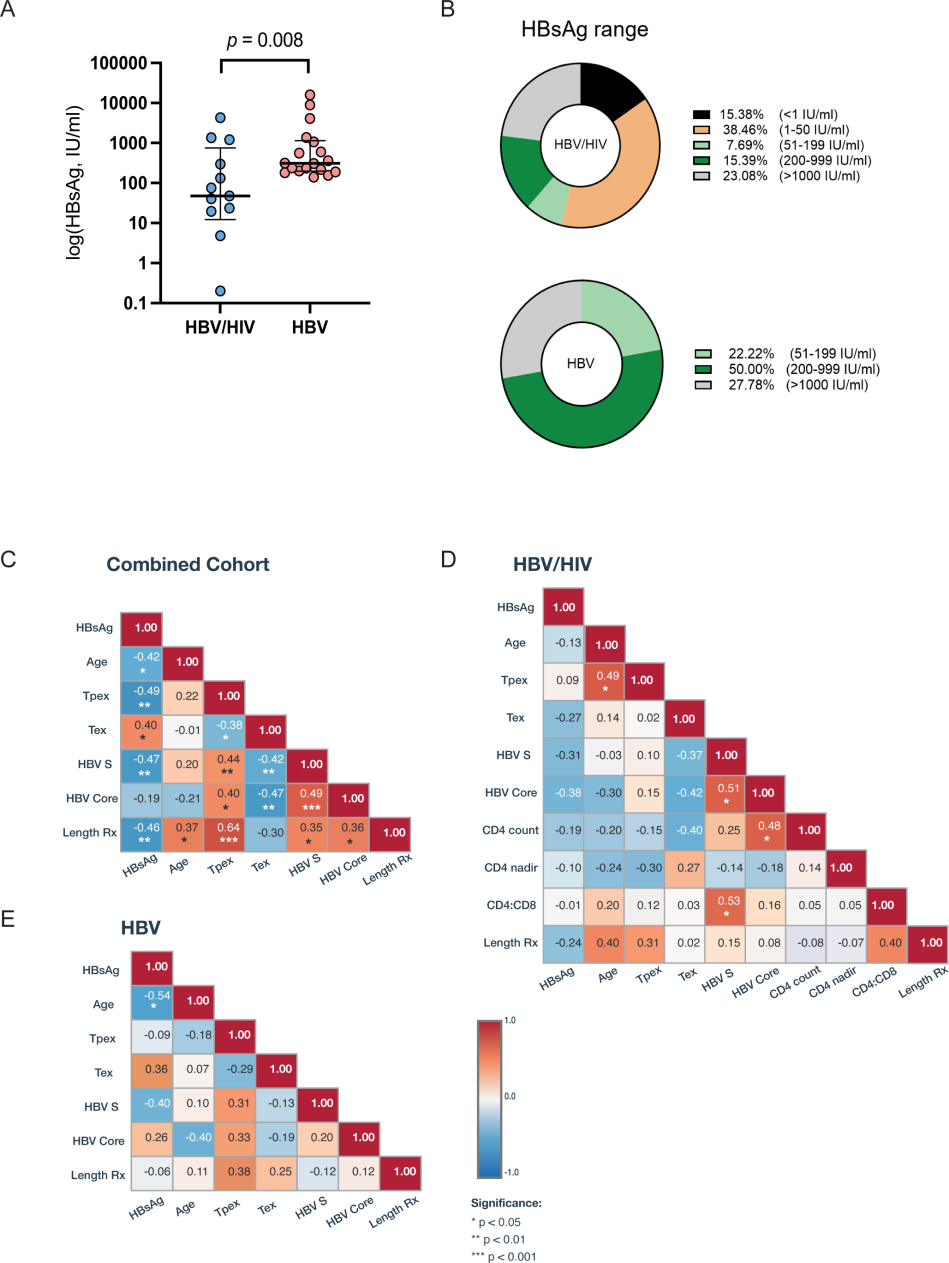
Virological parameters and correlation analysis. (**A**) HBsAg level quantification (log HBsAg, IU/mL) from individuals with HBV/HIV and HBV. Data shown as scatter plots with the group median and IQR indicated. (**B**) Proportion of HBV/HIV+or HBV+donors within a given range of HBsAg level (IU/mL). (**C**) Correlogram of individuals with HBV/HIV and HBV (combined cohort) utilised for phenotypic and functional characterisation. Group-specific correlograms for HBV/HIV group (**D**) and HBV group (**E**). Spearman r values shown from blue (−1) to red (1). HVsAg, hepatitis B surface antigen; HBV, hepatitis B virus.

Having established distinct phenotypic and functional differences in CD8^+^ T cell responses between patient groups, we examined potential virological factors that might explain these immunological differences. Quantitative HBsAg analysis revealed significantly lower levels in people with HBV/HIV co-infection compared with those with HBV mono-infection (figure 7A), in keeping with our recent observations.^14^ A greater percentage of people with HBV/HIV had HBsAg below 50 IU/mL, including some with levels below 1 IU/mL (figure 7B), potentially reflecting the longer duration of antiviral therapy in this group (average 14 years vs 4 years in mono-infection, online supplemental table 1).

When examining the entire cohort, we observed several associations (figure 7C). Circulating HBsAg levels showed negative associations with age, treatment duration and functional HBV surface-specific responses and Tpex frequencies (figure 7C). Tpex populations correlated positively with HBV-specific responses and treatment duration, while Tex populations showed inverse relationships with HBV functional responses (figure 7C). Across the entire cohort, treatment duration also positively associated with HBV-specific responses (figure 7C). However, stratified analyses by infection status revealed different patterns (figure 7D,E). In the HBV/HIV group, age positively correlated with Tpex frequencies and the CD4 count and CD4:CD8 ratio with HBV core-specific and surface-specific responses respectively (figure 7D). This aligns with our previous observations in treated HIV infection, where CD4:CD8 ratio was associated with improved antigen specific responses.^26^ In HBV mono-infection, a significant negative association was observed between age and HBsAg levels^37^ (figure 7E). Importantly, this stratified analysis revealed no significant within-group correlations between treatment duration and HBV-specific responses or CD8^+^ T cell subsets (figure 7D,E and online supplemental figure 8A–D). Similarly, within-group analyses showed no significant correlations between HBsAg levels and CD8^+^ T cell phenotypes or functional responses (figure 7D,E and online supplemental figure 8E–H). These findings suggest that the relationships observed reflect between-group differences rather than within-group relationships, with factors beyond treatment duration or HBsAg levels contributing to the distinct immunological profiles.

## Discussion

The goal of immune-based strategies for cHBV infection is to elicit functional and durable HBV-specific CD8^+^T cell responses that can bolster antiviral control. Our findings challenge the conventional understanding of immune responses in HBV/HIV co-infection by demonstrating that individuals with well-controlled HBV/HIV co-infection on long-term suppressive therapy display enhanced preservation of functionally competent HBV-specific CD8^+^ T cells compared with those with HBV mono-infection.

The increased frequency of TCF-1^+^CD127^+^PD-1^+^ (Tpex) CD8^+^ T cells observed in people with co-infection aligns with emerging evidence demonstrating the critical role of these cells in maintaining antiviral responses during human chronic infection.^38^,^42^ These memory-like CD8^+^ T cells, that occur early during infection,^43 44^ maintain proliferative capacity despite chronic antigen exposure and provide the proliferative burst following PD-1 pathway blockade.^16 17 32 35^ Our findings extend this concept to HBV/HIV co-infection, where we observe higher frequencies of Tpex cells and more robust HBV-specific responses than in HBV mono-infection. Furthermore, these results align with our recent work demonstrating enhanced NK cell ADCC in co-infection,^14^ suggesting a better-preserved immunological profile favouring viral control.

The transcriptional signature of global CD8^+^ T cells in HBV/HIV co-infection suggests adaptive mechanisms allowing CD8^+^ T cells to navigate a potentially more complex immunological landscape due to the presence of two chronic viral infections. Upregulation of TCR signalling and inhibitory pathway genes (eg, *CD160*, *TIGIT*, *PDCD1*) alongside genes involved in inflammation regulation (*CHI3L2*, *KLF10*) and mitochondrial function (*CMC1*) suggests a balanced immune response that prevents excessive inflammation while maintaining functional responses. Along these lines, PD-1 expression on Tpex virus-specific CD8^+^ T cells likely shields these populations from excessive TCR stimulation, preserving their stem-like properties.^15^ This interpretation is supported by our observation that these populations maintain high expression of BCL-2 and TCF-1, markers associated with memory potential and cellular longevity, despite chronic antigenic stimulation.^41^ In contrast, HBV mono-infection exhibited a profile skewed toward terminal exhaustion, with elevated TOX expression, that transcriptionally/epigenetically programmes CD8^+^ T cell exhaustion,^34^ and linked to CD8^+^ T cell dysfunction during cHBV.^45^

Our scRNA-seq analysis revealed global shifts in the CD8^+^ T cell landscape. Although these changes cannot be directly attributed to HBV-specific responses alone, we demonstrated that these differences extend to virus-specific populations. Direct ex vivo dextramer analysis confirmed that HBV-specific CD8^+^ T cells from people with co-infection maintain the Tpex phenotype observed globally. Our findings echo recent observations demonstrating that exhaustion profiles in global CD8^+^ T cells can predict functional capacity of HBV-specific responses.^6^ The apparent paradox of better T cell functionality in co-infection could be explained by several factors. Although we noted correlations across the entire cohort, stratified within-group analyses showed that neither treatment duration nor HBsAg levels alone predict CD8^+^ T cell profiles. While this could reflect limited variance in our study groups, it suggests the differences primarily reflect between group effects. Notably, the detection of correlations with immune parameters in HIV/HBV infection along with divergent age-related patterns suggests that HIV co-infection reshapes the immunological landscape. The combination of earlier treatment initiation at diagnosis, immune reconstitution quality, prolonged viral suppression (TDF therapy for a median of 16 years) and lower antigen burden in co-infection likely collectively contributes to the maintenance of functional T cell responses. Beyond suppressing viral replication, NUC therapy may also modulate liver inflammation and the immune microenvironment. Recent work demonstrated that TDF therapy reduces exhausted PD1+TOX+ CD8^+^ T cells with these changes correlating with reduced viral load transcription rather than HBsAg levels.^46^ While our peripheral blood observations cannot directly reflect intrahepatic changes, the prolonged TDF treatment in our co-infection cohort may similarly preserve the Tpex pool. Additionally, the immunological milieu created by HIV infection^47^ may modulate HBV-specific responses through altered cytokine profiles or reduced immune regulatory mechanisms. Our recent findings of enhanced proportions of adaptive/memory-like NK cells in HBV/HIV co-infection,^14^ with less inherent potential for excessive negative regulation of T cell responses,^48 49^ support this broader immune remodelling hypothesis, rather than simple linear relationships with any single factor.

Multiple studies have demonstrated that targeting the PD-1 pathway can enhance HBV-specific CD8^+^ T cell function^50^ and affect the HIV viral reservoir and virus-specific responses.^51 52^ The enhanced anti-PD-L1 responsiveness provides functional validation of the preserved Tpex populations in co-infection, as these cells specifically mediate the proliferative burst following checkpoint blockade. These findings suggest that people with co-infection, who maintain higher frequencies of checkpoint-responsive Tpex cells, may be optimal candidates for PD-1/PD-L1-targeted immunotherapeutic strategies. Similarly, approaches to target the TCF-1 pathway^53^ and enhance stemness through IL-15 signalling^54^ or TOX manipulation even after Tex establishment^55^ could therefore provide therapeutic opportunities to rewire Tex cells in chronic infections.

Several limitations of our study warrant consideration. As a primarily cross-sectional investigation conducted on peripheral blood, we cannot directly extrapolate our findings to hepatic tissue. The observed differences may be significantly influenced by our cohort characteristics, particularly regarding infection routes and treatment histories—factors that are inherently difficult to match, which may limit the generalisability of our findings. In Western settings, HBV/HIV co-infection typically occurs through sexual transmission in adulthood with prompt treatment initiation at diagnosis, while HBV mono-infection often results from vertical transmission with longer duration of untreated infection before therapy. These fundamental differences in immune priming and tolerance, along with timing of therapeutic intervention, further complicate direct comparisons. Additionally, our analysis focused on surface and core HBV-specific responses; extending this to other antigens would provide a more comprehensive understanding of virus-specific responses, as exhaustion profiles differ based on targeted antigens.^6 41^

The substantially different treatment durations in our cohort reflect inherent clinical realities of these populations, precluding definitive separation of treatment-related from infection-specific immune effects. Future longitudinal studies tracking both groups from treatment initiation are needed to examine how these immunological differences evolve over time in response to therapy and correlate with clinical outcomes. However, current treatment guidelines (immediate ART for HIV vs criteria-based treatment for HBV mono-infection) present practical challenges for matched comparisons. Additional work beyond the scope of this study will aim to provide more mechanistic insights into the maintenance of Tpex populations in co-infection that could identify novel targets for enhancing immune function.

Despite these limitations, our study provides the first evidence that in individuals with sustained well-controlled HBV/HIV co-infection in the current ART era, CD8^+^ T cell responses are more robust and retain stem-like properties that support ongoing antiviral function. These findings underscore the potential benefits of early antiretroviral intervention and highlight TCF-1-expressing stem-like CD8^+^ T cells as a promising target for immunotherapeutic strategies aimed at achieving FC in cHBV infection.

## Supplementary material

10.1136/gutjnl-2025-335461 online supplemental file 1

10.1136/gutjnl-2025-335461 online supplemental file 2

10.1136/gutjnl-2025-335461 online supplemental file 3

## Data Availability

Data are available in a public, open access repository. All data relevant to the study are included in the article or uploaded as supplementary information.
