# Supplementary material for: Stem-like CD8+ T cells preserve HBV-specific responses in HBV/HIV co-infection
Source: Gut. 2025 Dec 4;75(7):e335461. doi: 10.1136/gutjnl-2025-335461 (PMC12848322; doi:10.1136/gutjnl-2025-335461)
Supplement: online supplemental file 1 [file gutjnl-75-7-s001.docx]

**Supplementary Information:**

**Stem-like CD8+ T cells preserve HBV-specific responses in HIV/HBV co-infection**

Jay Preechanukul^1^, Aljawharah Alrubayyi^2^, Bo Sun^3^, Edward Arbe-Barnes^1^, Jonida Kokici^1^, Frances Gorou^1^, Sarun Prasitdumrong^1^, Kelly A.S. da Costa^1^, Natasha Fisher-Pearson^2^, Noshin Hussain­^1^, Stephanie Kucykowicz^1^, Indrajit Ghosh^4^, Fiona Burns^5,6^, Sabine Kinloch^5^, Pedro Simoes^5^, Sanjay Bhagani^1,7^, Patrick T F Kennedy^8^, Mala K Maini^1^, Rachael Bashford-Rogers^9^, Upkar S Gill^8^, Dimitra Peppa^1,4,5^

**Supplementary Figure 1. Transcriptomic profiles of CD8 T cell subsets in HBV monoinfection vs. HBV/HIV co-infection. (A)** SingleR annotations using the Monaco Immune Dataset as reference **(B)** Alluvial plot showing proportion consensus between annotations and SingleR assignments. **(C)** Boxplots of cytotoxicity scores across control (CTRL), mono-infection (HBV) and co-infection (HBV/HIV). **(D)** Expression heatmap showing activity of gene programmes across CD8 cell subsets. **(E)** Top genes related to each dimension. Abbreviations: CTRL, Control; HBV, Hepatitis B Virus; HIV, Human immunodeficiency virus; UMAP, uniform manifold approximation and projection; DR, Dimensionality reduction.

**Supplementary Figure 2.** **DR17-associated gene expression in CD8 T cells across HBV mono-infection and HBV/HIV co-infection. (A)** Pseudobulk expression (log₁₊ counts per million, CPM) of top DR17-associated genes in CD8 T cells from individuals with HBV mono-infection and HBV/HIV co-infection; none reached significance individually in two-sided Mann–Whitney U tests after Benjamini–Hochberg correction. **(B)** UMAP visualisation showing expression of Tpex score-defining genes from n = 11 individuals (HBV, n=6; HIV/HBV co-infection, n=5). Abbreviations: HBV, Hepatitis B Virus; HIV, Human immunodeficiency virus; UMAP, uniform manifold approximation and projection.

**Supplementary Figure 3. Characterisation of CD8^+^ T cell subsets in HBV/HIV, HBV, and HIV infection**. **(A)** Representative flow plots of gating strategy of CD8^+^ T cell identification. **(B)** Summary data of frequencies of global CD8^+^ T cells in individuals with HBV/HIV (n = 19), HBV (n = 20) and HIV (n = 21). **(C)** Representative flow plots with pie charts representing the proportion of global CD8^+^ T cells with a CD45RA^+^/CCR7^+^ naïve, CD45RA^−^/CCR7^+^ central memory (TCM), CD45RA^−^/CCR7^−^ effector memory (TEM), and CD45RA^+^/CCR7^−^ terminally differentiated effector memory (TEMRA) phenotypes and **(D)** summary data from HBV/HIV+ (n=19), HBV+(n=20) and HIV+ (n = 21) donors. Bar charts show the median value with interquartile range and each dot represents one donor in a group. Statistical significance was assessed by Kruskal-Wallis with Dunn's multiple comparison test (p < 0.05). **(E)** Summary data of frequency of PD-1^+^ CD8^+^ cells within CEF, HBV S, HBV core or HIB gag -specific populations from individuals with HBV/HIV, HBV and HIV. Statistics were performed using a two-tailed Wilcoxon matched-pairs signed-rank test (*p* < 0.05).

**Supplementary Figure 4**. **Phenotypic characteristics of CD8^+^ T cells in HBV/HIV, HBV and HIV infection.** Representative flow plots and summary data of the relative frequency of **(A)** PD-1^+^, **(B)** CD127^+^, **(B)** HLA-DR^+^CD38^+^ and **(C)** CD127^+^, on CD8^+^ T cells from HBV/HIV+ (n=19), HBV+(n=20) and HIV+ (n = 21) donors. **(D)** Representative flow cytometric histogram plots and summary data of TOX MFI from HBV/HIV+ (n = 19), HBV+ (n = 20) and HIV+ (n = 21) donors. Bar charts show the median value with interquartile range and each dot represents one donor in a group. Statistical significance was assessed by Kruskal-Wallis with Dunn's multiple comparison test (*p* < 0.05).

**Supplementary Figure 5**. **Phenotypic characteristics of Tpex and Tex CD8^+^ T cells in HBV/HIV, HBV and HIV infection. (A)** Representative flow plots and **(B)** pie charts representing the proportion of CD8^+^Tpex and Tex with a CD45RA^+^/CCR7^+^ naïve (TN), CD45RA^−^/CCR7^+^ central memory (TCM), CD45RA^−^/CCR7^−^ effector memory (TEM), and CD45RA^+^/CCR7^−^ terminally differentiated effector memory (TEMRA) phenotype. **(C)** Representative flow plots of Tpex and Tex profiles identified by expression of TCF-1, CD127 and granzyme B in peripheral blood, and **(D)** summary data from HBV/HIV+ (n = 19), HBV+ (n = 20), and HIV+ (n = 20) donors. Bar charts show the median value with interquartile range and each dot represents one donor in a group. Statistical significance was assessed by Kruskal-Wallis with Dunn's multiple comparison test (*p* < 0.05).

**Supplementary Figure 6**. **Phenotypic characterisation of HBV and HIV-specific CD8^+^ T cells in HBV/HIV, HBV, and HIV infection. (A)** a representative flow plots and **(B)** pie charts representing the proportion of antigen-specific CD8^+^ T cell with a CD45RA^+^/CCR7^+^ naïve, CD45RA^−^/CCR7^+^ central memory (TCM), CD45RA^−^/CCR7^−^ effector memory (TEM), and CD45RA^+^/CCR7^−^ terminally differentiated effector memory (TEMRA) phenotype from HBV/HIV+ (n = 20), HBV+ (n = 20), and HIV+ (n = 21) donors against overlapping HBV-S, HBV-core and HIV-gag peptides. **(C)** Representative flow plots and summary data **(D)** of the expression of PD-1 within CEF, HBV-S, HBV-core or HIV-gag -specific CD8^+^ T cells from individuals with HBV/HIV, HBV and HIV. Statistics were performed using a two-tailed Wilcoxon matched-pairs signed-rank test (p < 0.05).

**Supplementary Figure 7.**  **Ex vivo characterisation of CMV pp65- and HIV gag-specific CD8^+^ T cells. (A)** Representative flow plots of identification of virus-specific CD8^+^ T cells via dextramer staining directly *ex vivo* **(B)** summary data of CD127/PD1 co-expression analysis of CMV pp65- and HIV gag-specific CD8^+^ T cells derived from individuals with HBV/HIV (n = 6). Summary data of expression of **(C)** TCF-1 and **(D)** BCL-2 with respect to the CD127/PD1 subsets of CMV pp65- and HIV gag-specific CD8^+^T cells and summary data from HBV/HIV+ (n = 6). Bar charts show the median value with interquartile range. Statistics were performed using a two-tailed Wilcoxon matched-pairs signed-rank test (*p* < 0.05)

**Supplementary Figure 8. Stratified correlation analyses by infection status.** (**A-B**) Treatment duration versus HBV surface and core-specific CD8+ T cell responses. (**C-D**) Treatment duration versus Tpex and Tex CD8+ T cell frequencies. (**E-F**) Log HBsAg levels versus HBV surface and core-specific CD8+ T cell responses. (**G-H**) Log HBsAg levels versus Tpex and Tex CD8+ T cell frequencies. Each dot represents one individual. Lines show linear regression fits. Spearman correlation coefficients (r) and p-values are shown for each group. People with HBV/HIV co-infection are shown in blue and HBV mono-infection in red.

**SUPPLEMENTARY TABLES**

**Supplementary Table 1. Cohort characteristics for all participants.** All patients included are classed as non-viraemic where HBV DNA and / or HIV viral load is below the limit of detection of the assay performed as part of clinical assessment.

|  |  | HBV/HIV | HBV | HIV |
| --- | --- | --- | --- | --- |
| Group Size | n | 20 | 20 | 21 |
| Age | Median (IQR) | 57 (30-72) | 44 (30-83) | 52 (34-72) |
| Sex | Male (n) | 16 | 18 | 17 |
|  | Female (n) | 4 | 2 | 4 |
| Ethnicity | White n (%) | 10 (50.0%) | 6 (30.0%) | 12 (57.14%) |
|  | BAME n (%) | 10 (50.0%) | 14 (70.0%) | 9 (42.86%) |
| HBV Parameters | Viral load  (DNA Iu/ml) | BLD* | BLD* | N/A |
|  | eAg status | Positive – 3  Negative - 17 | Positive – 4  Negative – 16 | N/A |
|  | ALT, Median (range) | 29 (10-90) | 32 (18 –80) | N/A |
|  | HBsAg (IU/ml), Median (range) | 61.45  (0.2-4274) | 237.3  (141.1-10953) | N/A |
| HIV Parameters | Viral load (RNA copies/ml) | BLD* | N/A | BLD* |
|  | CD4 count, Median (range) | 468.50 (81 - 1024) |  | 571 (177-1213) |
|  | CD4 nadir, Median (range) | 146.50 (2.00-475.00 |  | 396.50 (13.00-610.00) |
|  | CD4:CD8 ratio Median (range) | 0.87 (0.36 – 2.88) |  | 0.90 (0.29-3.05) |
| Median Treatment Length and range | | Tenofovir based cART  16 (years (3-21) | NUC^+^  2.15 years (0.5-11) | Tenofovir based cART  6 years (2-18) |

**BLD – Below Limit of Detection during routine blood testing in clinic.*

*+ NUC - Nucleotide analogues (e.g. Tenofovir n = 16, Entecavir n = 4)*

**Supplementary Table 2. Cohort characteristics for participants analysed by scRNA.** All patients selected from the cohort were classed as non-viraemic where HBV DNA and / or HIV viral load is below the limit of detection during routine clinical assessment analysis.

| **PID** | **Ethnicity** | **Age** | **Sex** | **HBV serology** | **HBsAg**  **(IU/ml)** | **ALT**  **(IU/L)** | **HBV DNA** | **HIV RNA** | **CD4**  **count** | **Total**  **CD8**  **(scRNA)** |
| --- | --- | --- | --- | --- | --- | --- | --- | --- | --- | --- |
| HBV-1 | BAME | 44 | M | eAg- | 279 | 105 | BLQ | NA | NA | 1586 |
| HBV-2 | White | 37 | M | eAg- | NA | 69 | BLQ | NA | NA | 579 |
| HBV-3 | BAME | 46 | M | eAg+ | 1032 | 51 | BLQ | NA | NA | 1528 |
| HBV-4 | White | 56 | F | eAg- | 2544 | 53 | BLQ | NA | NA | 1098 |
| HBV-5 | BAME | 35 | M | eAg- | 7765 | 17 | BLQ | NA | NA | 1909 |
| HBV-6 | BAME | 55 | M | eAg- | 35 | 10 | BLQ | NA | NA | 1592 |
| HBV/HIV-1 | BAME | 44 | M | eAg- | 208 | 42 | BLQ | BLQ | 903 | 943 |
| HBV/HIV-2 | White | 69 | M | eAg- | 1.92 | 41 | BLQ | BLQ | 434 | 2620 |
| HBV/HIV-3 | BAME | 42 | M | eAg+ | 75 | 23 | BLQ | BLQ | 556 | 1622 |
| HBV/HIV-4 | White | 57 | M | eAg- | 133 | 21 | BLQ | BLQ | 449 | 2207 |
| HBV/HIV-5 | White | 32 | M | eAg- | NA | 36 | BLQ | BLQ | NA | 2591 |

**Supplementary Table 3. Biological interpretation for all DRVI-derived programmes**

| **DR Programme** | **Programme Annotation** |
| --- | --- |
| **DR1−** | Cytotoxic / terminal effector (innate-like) |
| **DR1+** | Naïve/central-memory / stem-like |
| **DR2+** | Effector / NK-like properties |
| **DR2−** | IL7R⁺ precursor / memory-biased |
| **DR3+** | Tissue-resident memory / NK-like |
| **DR4+** | CD8 lineage |
| **DR4−** | Tc17/MAIT-like effector cells |
| **DR5+** | Central-memory/survival signalling |
| **DR6−** | Terminal cytotoxic |
| **DR6+** | Housekeeping / Translation |
| **DR7−** | Conventional T adhesion / Migration |
| **DR7+** | Checkpoint Receptor–Enriched NK-like |
| **DR8+** | GZMK⁺ effector-memory |
| **DR9+** | Follicular helper-like |
| **DR10−** | Cytoskeleton/adhesion & motility |
| **DR10+** | Immediate-early activation |
| **DR11−** | IFN / inflammatory |
| **DR11+** | Mitochondrial |
| **DR12+** | Tissue-tropic TRM-like cytotoxic |
| **DR13−** | NR4A/activation–anergy module |
| **DR14+** | Acute activation / cytokine response |
| **DR16+** | Cycling/proliferation & DNA repair |
| **DR17+** | Progenitor exhausted |
| **DR20+** | PLAC8⁺ stress/inflammation |
| **DR21−** | Naïve/central-memory & chromatin-stemness |
| **DR22−** | IFN/mitochondrial stress + motility |
| **DR22+** | Ambient Red Blood Cell RNA |
| **DR23−** | Ambient Non-T |
| **DR26+** | N/A – too few genes |
| **DR27+** | Chromatin packaging/quiescent |
| **DR28+** | N/A – too few genes |
| **DR31+** | Inflammatory chemokine |
| **DR70+** | N/A – too few genes |

**Supplementary Table 4. Antibody table**

| **Antibody** | **Supplier** | **Cat no.** | **Clone** |
| --- | --- | --- | --- |
| BV510 anti-human CD14 | Biolegend | 301842 | M5E2 |
| BV510 anti-human CD19 | Biolegend | 302242 | HIB19 |
| Alexa Fluor 700 anti-human granzyme B | BD Bioscience | 560213 | GB11 |
| BV650 anti-human CD3 | Biolegend | 317324 | OKT3 |
| BV711 anti-human CD8 | Biolegend | 301044 | RPA-T8 |
| PE/Dazzle594 anti-human CD4 | Biolegend | 300548 | RPA-T4 |
| BV785 anti-human CD38 | Biolegend | 303530 | HIT2 |
| BV421 anti-human PD-1 | Biolegend | 329920 | EH12.2H7 |
| Alexa Fluor 700 anti-human CD45RA | Biolegend | 304120 | HI100 |
| FITC anti-human TNF-α | Biolegend | 502906 | Mab11 |
| APC anti-human IFN-γ | Biolegend | 506510 | B27 |
| PerCP eFluor710 anti-human IL-2 | eBioscience | 46702942 | MQ1-17H12 |
| PE-Cy7 anti-human CD154 | Biolegend | 310832 | 24-31 |
| Live/Dead Aqua | ThermoFisher Scientific | L34957 | N/A |
| BB700 anti-human CD4 | BD Bioscience | 566393 | SK3 |
| PE-Cy5 anti-human HLA-DR | BD Bioscience | 562007 | G46-6 |
| PE Texas Red anti-human TIM-3 | Biolegend | 345034 | F38-2E2 |
| PE anti-human LAIR-1 | Abcam | AB269308 | NKTA255 |
| APC-Cy7 anti-human CCR7 | Biolegend | 353212 | G043H7 |
| PE-Cy7 anti-human CD45RA | Biolegend | 304126 | HI100 |
| BV650 anti-human CD127 | Biolegend | 351326 | A019D5 |
| BV605 anti-human CD3 | Biolegend | 317322 | OKT3 |
| FITC anti-human TCF-1 | Cell Signaling Technology | 6444S | C63D9 |
| APC- anti-human TOX | Miltenyi Biotec | 130118474 | REA473 |

**Supplementary Table 5. Key Peptides and Chemicals,**

| **Antigen** | **Supplier** | **Identifier** |
| --- | --- | --- |
| PepMix HBV (Capsid protein) | JPT Peptide technologies | PM-HBV-CP |
| PepMix HBV (LEP) Ultra | JPT Peptide technologies | PM-HBV-LEPULTRA |
| PepMix HIV-1 (GAG) Ultra | JPT Peptide technologies | PM-HIV-GAG |
| CEF | Miltenyi Biotec | 130-098-426 |
| Brefeldin A | eBioscience | 00-4506-51 |
| BD Cytofix/Cytoperm™ Fixation/Permeabilization Solution Kit | BD Biosciences | 554714 |
| Foxp3/TF Staining  Buffer Set | eBioscience | 00-5523-00 |
|  |  |  |
