# Supplementary material for: Stem-like CD8+ T cells preserve HBV-specific responses in HBV/HIV co-infection
Source: Gut. 2025 Dec 4;75(7):e335461. doi: 10.1136/gutjnl-2025-335461 (PMC12848322; doi:10.1136/gutjnl-2025-335461)

Supplementary Fig 1

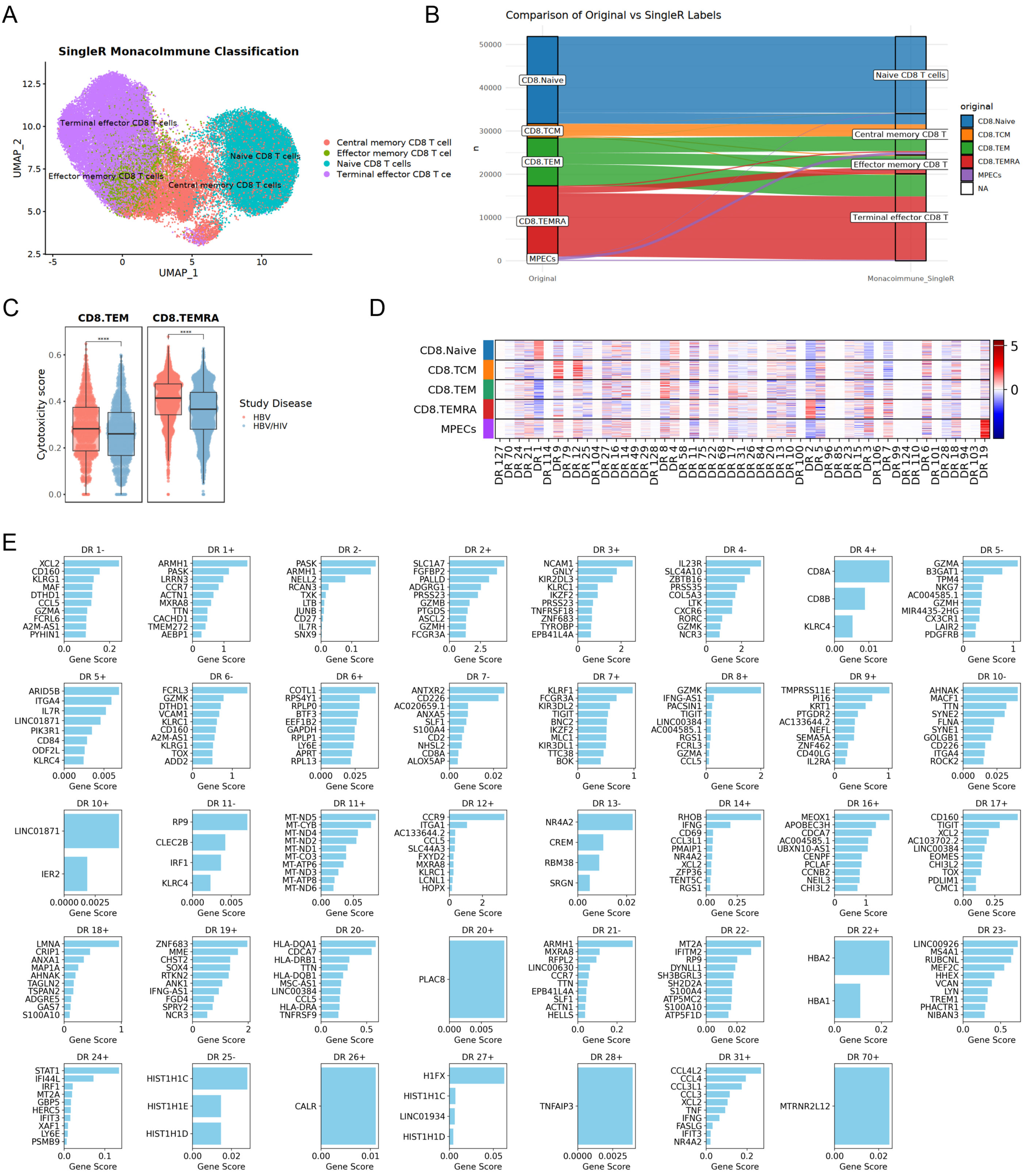

Supplementary Figure 2

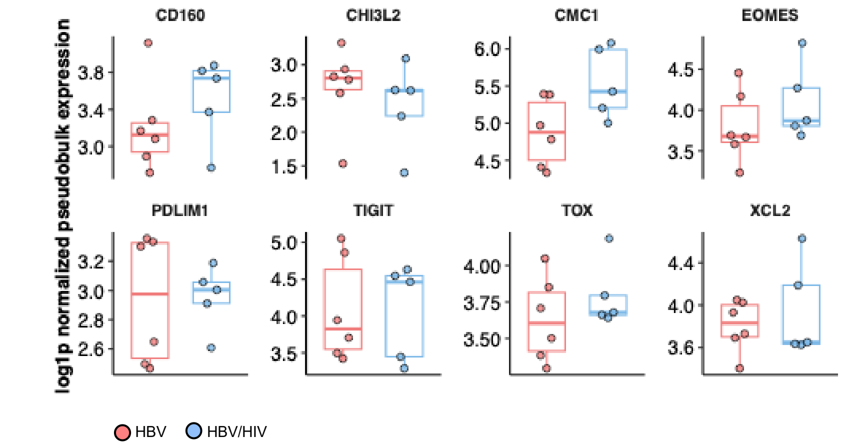

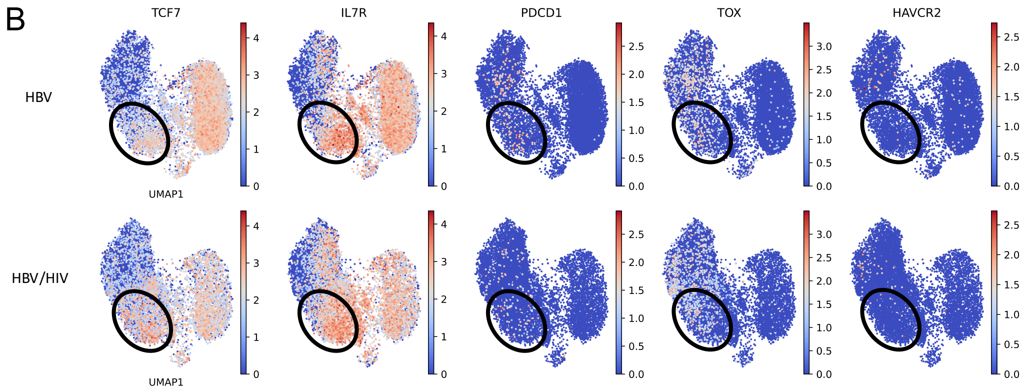

Supplementary Fig 3

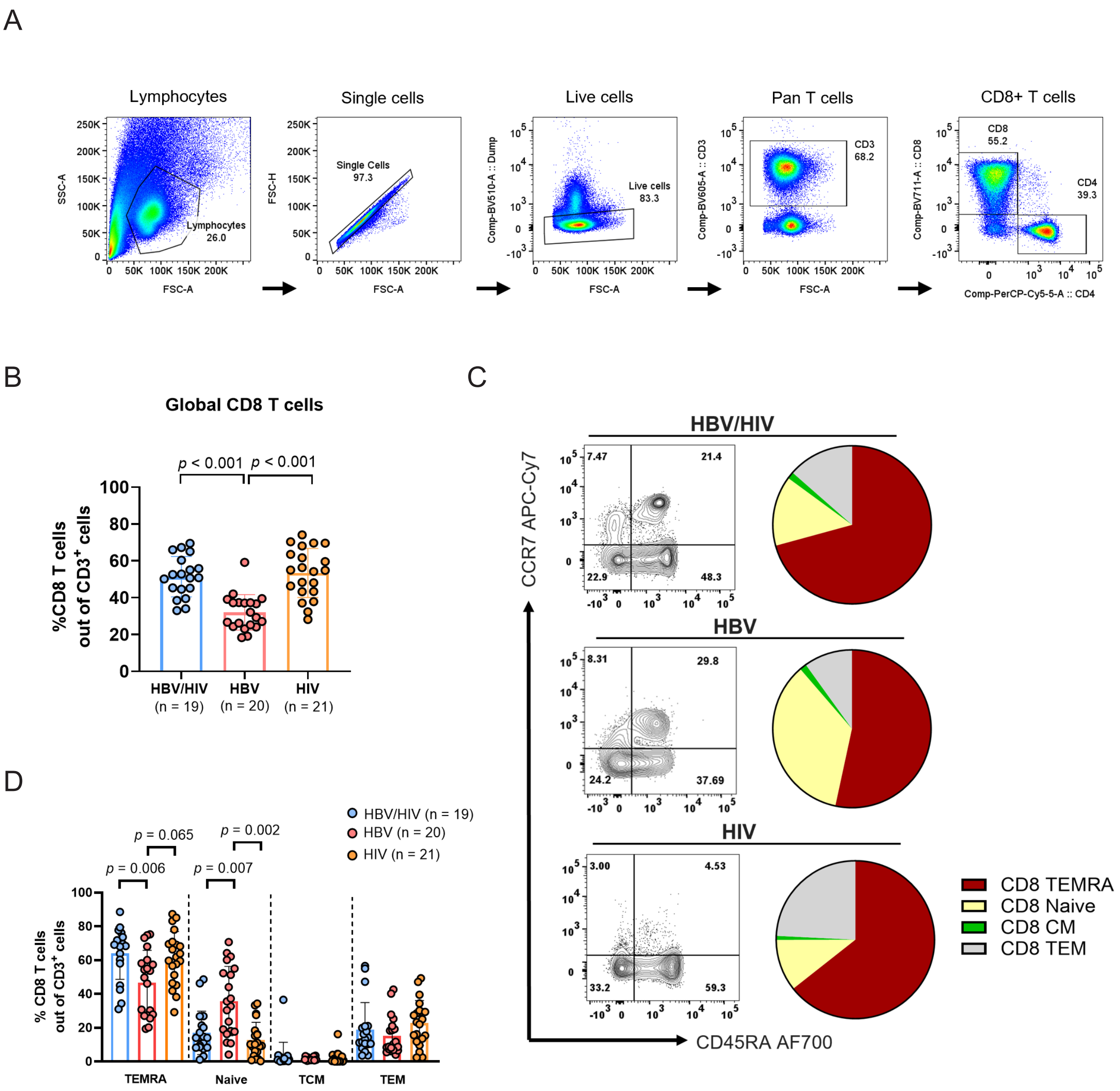

Supplementary Fig 4

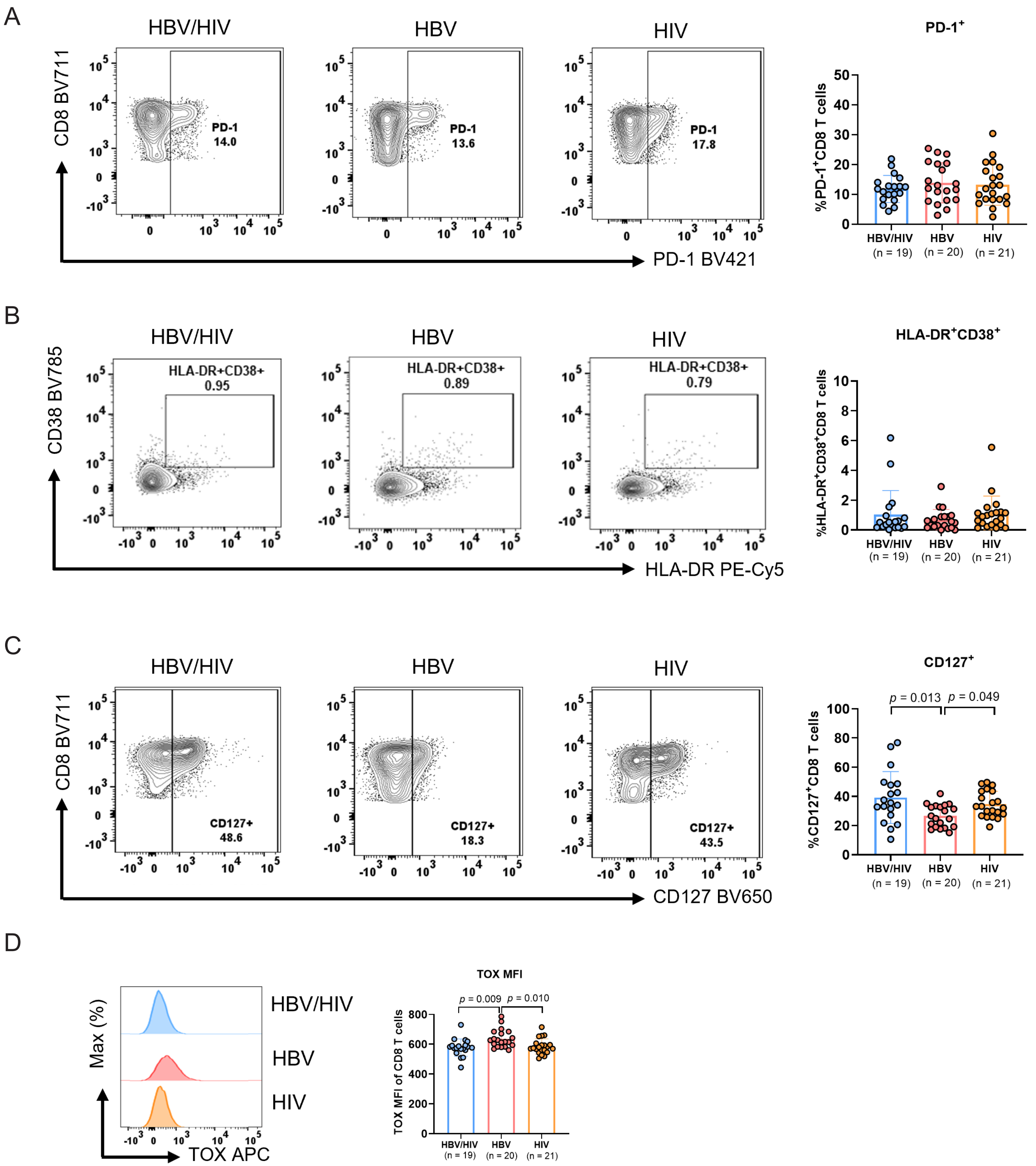

Supplementary Fig 5

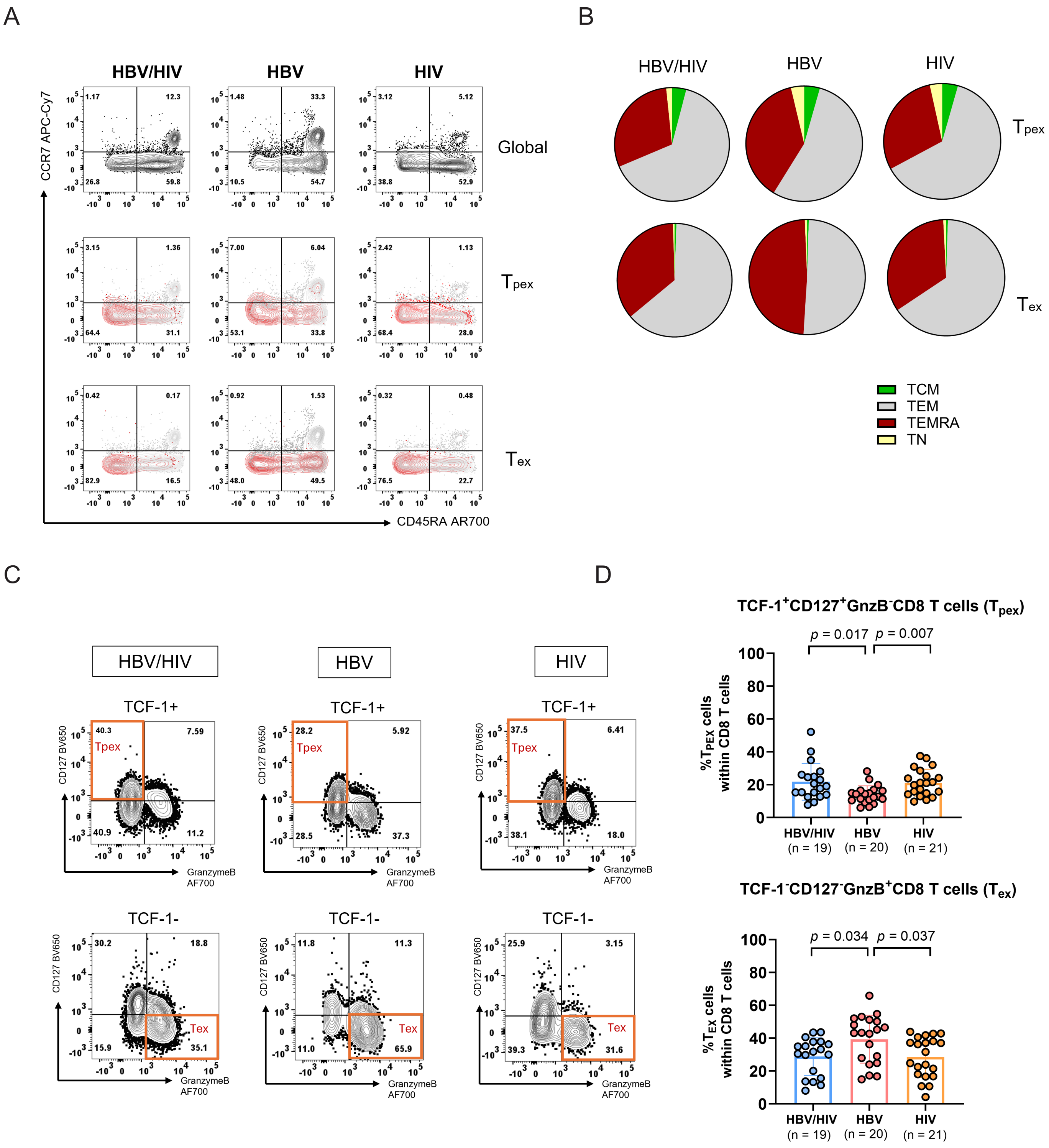

Supplementary Fig 6

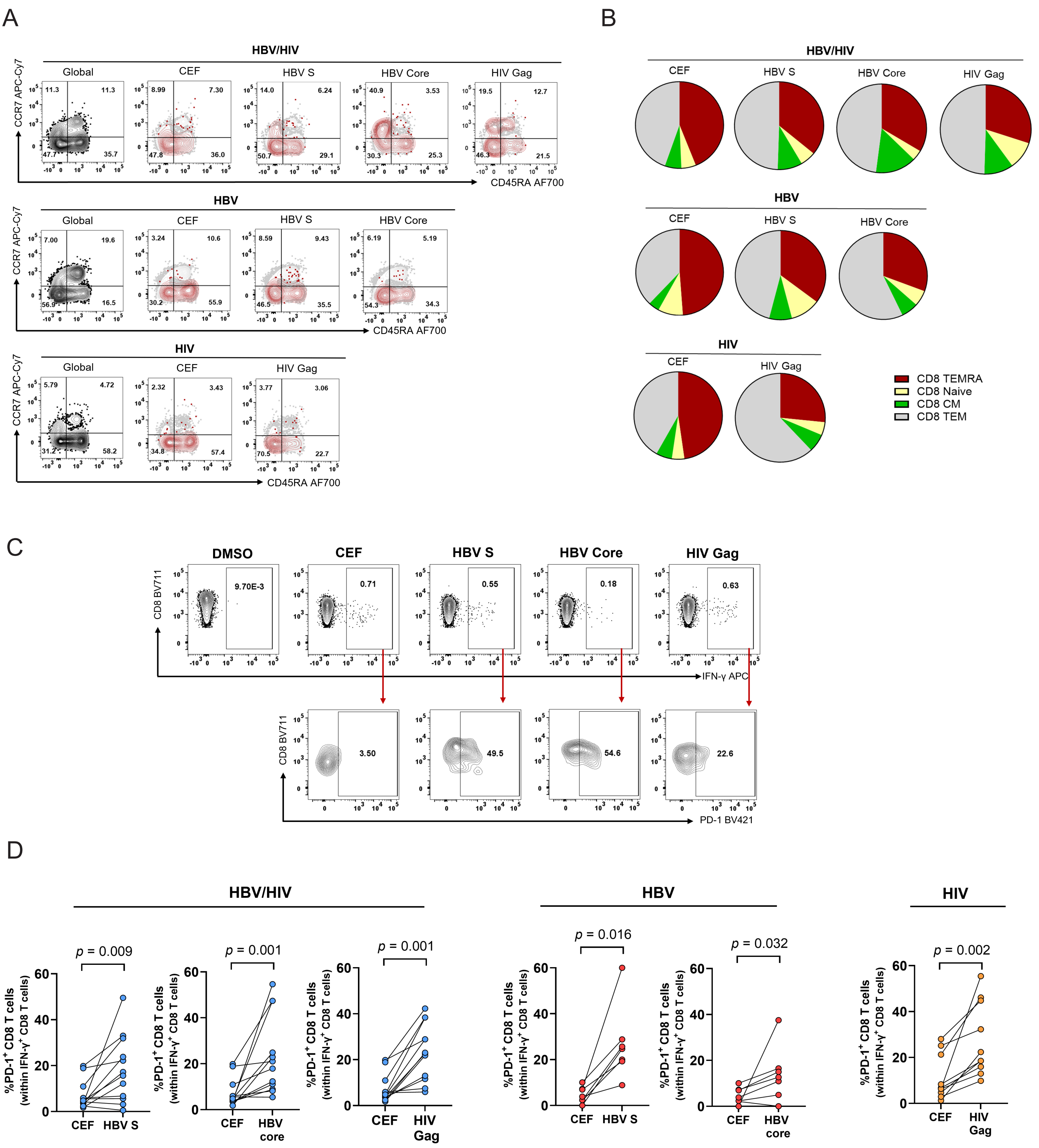

Supplementary Fig 7

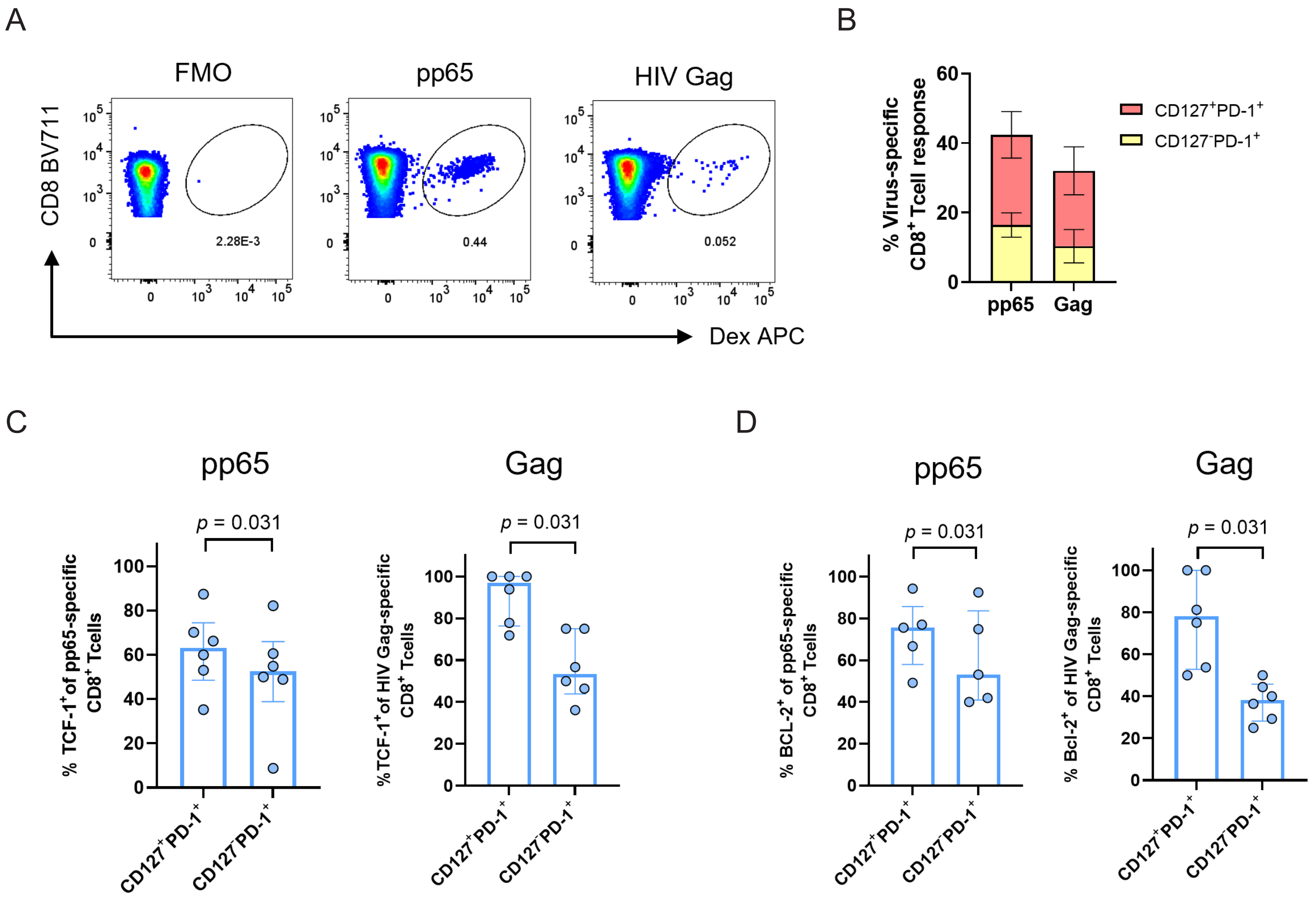

Supplementary Fig 8

CD8 T cell vs Length of therapy

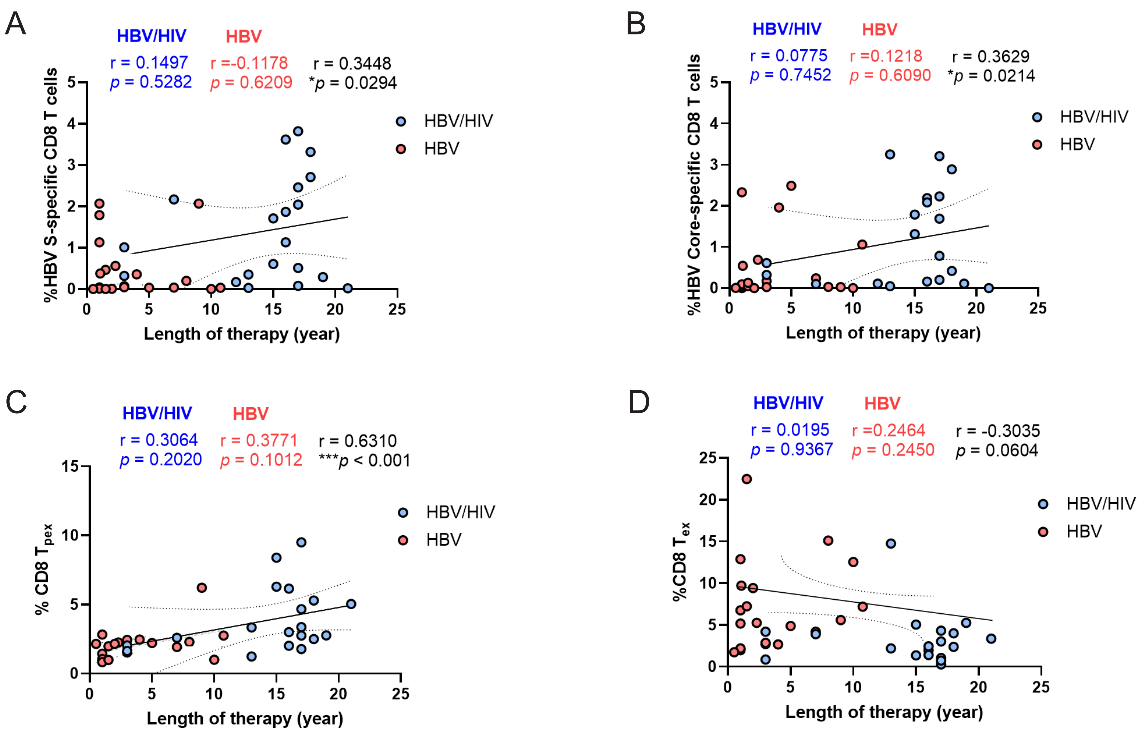

CD8 T cell vs HBsAg level

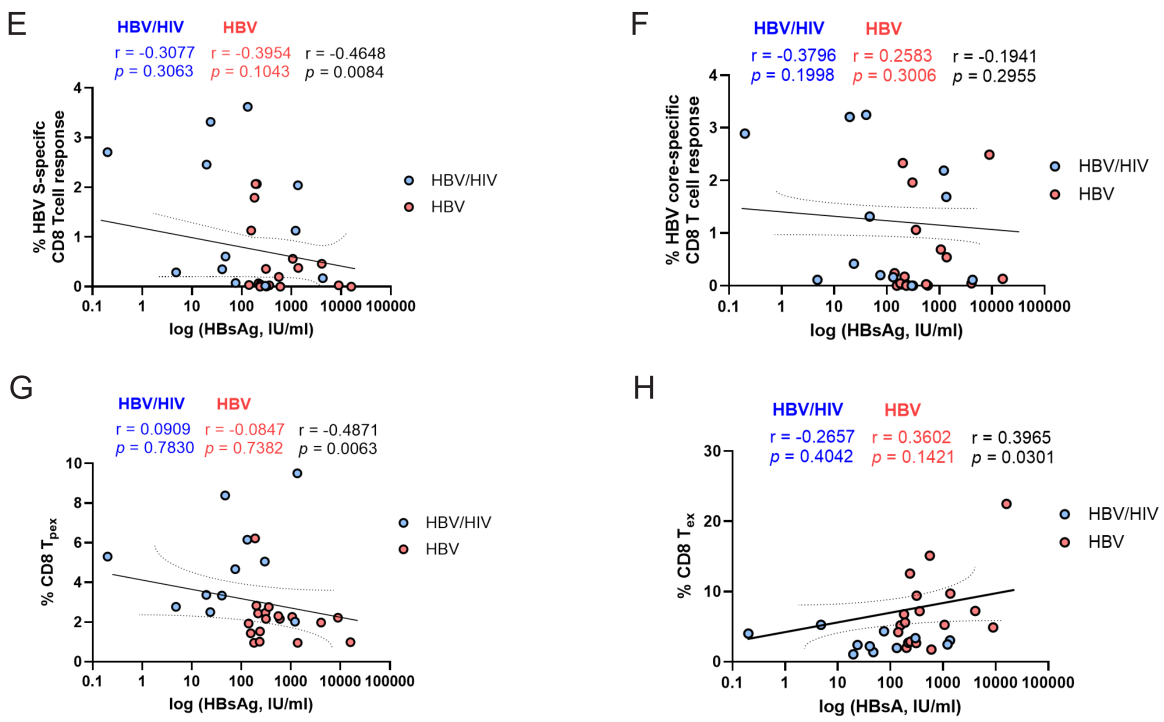

Supplement: online supplemental file 2 [file gutjnl-75-7-s002.pdf]
