# Supplementary material for: Stem-like CD8+ T cells preserve HBV-specific responses in HBV/HIV co-infection
Source: Gut. 2025 Dec 4;75(7):e335461. doi: 10.1136/gutjnl-2025-335461 (PMC12848322; doi:10.1136/gutjnl-2025-335461)
Supplement: online supplemental file 3 [file gutjnl-75-7-s003.pptx]

## Slide 1
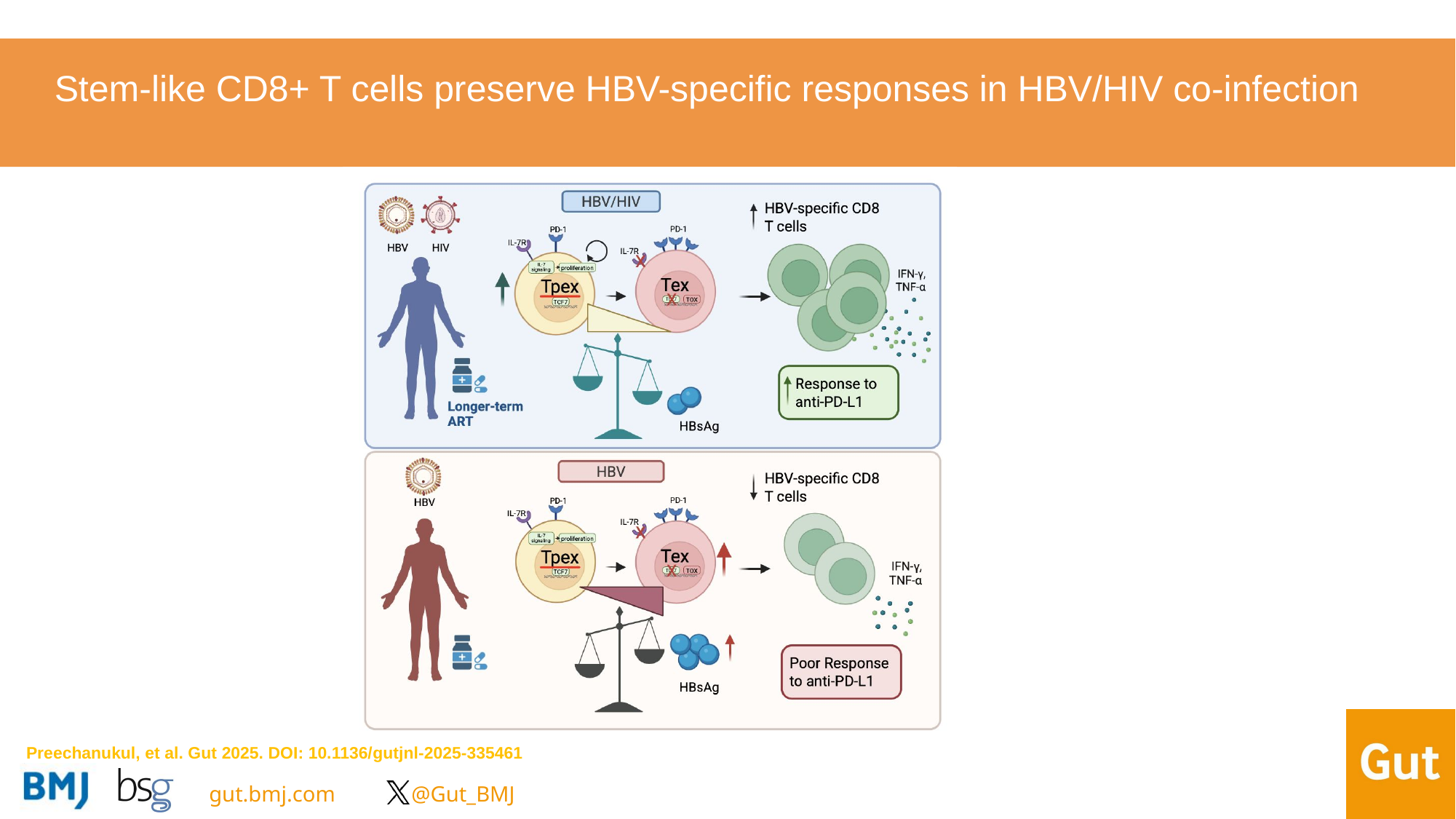

Stem-like CD8+ T cells preserve HBV-specific responses in HBV/HIV co-infection
Preechanukul, et al. Gut 2025. DOI: 10.1136/gutjnl-2025-335461
gut.bmj.com
@Gut_BMJ
